# The financial burden of noncommunicable diseases from out-of-pocket expenditure in sub-Saharan Africa: a scoping review

**DOI:** 10.1093/heapro/daae114

**Published:** 2024-09-17

**Authors:** Adelakun Odunyemi, Md Tauhidul Islam, Khurshid Alam

**Affiliations:** Murdoch Business School, Management & Marketing Department, Murdoch University, 90 South Street, Murdoch, Perth, Western Australia 6150, Australia; Hospitals Management Board, Clinical Department, Alagbaka, Akure 340223, Ondo State, Nigeria; Murdoch Business School, Management & Marketing Department, Murdoch University, 90 South Street, Murdoch, Perth, Western Australia 6150, Australia; Murdoch Business School, Management & Marketing Department, Murdoch University, 90 South Street, Murdoch, Perth, Western Australia 6150, Australia

**Keywords:** noncommunicable diseases, out-of-pocket catastrophic health expenditures, impoverishment, coping strategies, crowding-out effect on consumption, unmet needs, scoping review, sub-Saharan Africa

## Abstract

The growing financial burden of noncommunicable diseases (NCDs) in sub-Saharan Africa (SSA) hinders the attainment of the sustainable development goals. However, there has been no updated synthesis of evidence in this regard. Therefore, our study summarizes the current evidence in the literature and identifies the gaps. We systematically search relevant databases (PubMed, Scopus, ProQuest) between 2015 and 2023, focusing on empirical studies on NCDs and their financial burden indicators, namely, catastrophic health expenditure (CHE), impoverishment, coping strategies, crowding-out effects and unmet needs for financial reasons (UNFRs) in SSA. We examined the distribution of the indicators, their magnitudes, methodological approaches and the depth of analysis. The 71 included studies mostly came from single-country (*n* = 64), facility-based (*n* = 52) research in low-income (*n* = 22), lower-middle-income (*n* = 47) and upper-middle-income (*n* = 10) countries in SSA. Approximately 50% of the countries lacked studies (*n* = 25), with 46% coming from West Africa. Cancer, cardiovascular disease (CVD) and diabetes were the most commonly studied NCDs, with cancer and CVD causing the most financial burden. The review revealed methodological deficiencies related to lack of depth, equity analysis and robustness. CHE was high (up to 95.2%) in lower-middle-income countries but low in low-income and upper-middle-income countries. UNFR was almost 100% in both low-income and lower-middle-income countries. The use of extreme coping strategies was most common in low-income countries. There are no studies on crowding-out effect and pandemic-related UNFR. This study underscores the importance of expanded research that refines the methodological estimation of the financial burden of NCDs in SSA for equity implications and policy recommendations.

**Contribution to Health Promotion**

- Monitoring financial protection for noncommunicable diseases is important for identifying coverage gaps and targeting health promotion and prevention policies.
- Financial protection is crucial for achieving universal health coverage and promoting healthy living and well-being in sub-Saharan Africa.
- The study highlights major gaps in studies evaluating inequities associated with the financial burden of NCDs in sub-Saharan Africa.
- This will promote health equity and make health promotion services more affordable and accessible for vulnerable populations.
- This evidence in the indicators of financial protection can guide the design of health financing reforms to better support health promotion activities.

## BACKGROUND

Noncommunicable diseases (NCDs) account for ~71% of annual global mortality, and >80% of these deaths occur in low- and middle-income countries (LMICs) (WHO, 2018a). In sub-Saharan Africa (SSA), NCDs are the second most common cause of mortality and morbidity, accounting for almost 35% of deaths. Over the past two decades, the burden of NCDs in the SSA region has increased faster than elsewhere (Roser and Ritchie, 2016; Hunter-Adams *et al*., 2017). It has also been predicted that by 2030, they will overtake infectious diseases as the leading cause of death in SSA (WHO Regional Office for Africa, 2019). Africa has continued with its pre-pandemic NCD mortality level because of its low COVID-19-related mortality (WHO, 2024). Rapid urbanization, increasing ageing population, adoption of Westernized diets and other high-risk social behaviours, such as increased tobacco and alcohol use and lack of physical activity, are important drivers of the NCD epidemic in SSA (Nyirenda, 2016; Gyasi *et al*., 2020). The situation is worsened by the poor implementation of NCD-related policies in SSA and competing priorities like infectious diseases and maternal and child health (Allen *et al*., 2023). Health systems in most SSA countries are underfunded, have severely inadequate human resources and are ill-equipped to handle the rising burden of NCDs effectively, which has led to poor access to and increased costs of NCD management (WHO Regional Office for Africa, 2023; WHO, 2024). The COVID-19 pandemic has diverted focus from NCDs and worsened access to essential health services, including NCD care (WHO, 2023b; Odunyemi *et al*., 2024). These factors have multiplicative effects on the burden of NCDs in SSA.

Along with this high prevalence, the financial burden of NCDs poses a significant threat to the attainment of the United Nations Sustainable Development Goals (SDGs) in SSA by 2030 (Niessen *et al*., 2018; WHO, 2024). SSA is at an increasing risk of financial burden because the average proportion of total health expenditure spent as out-of-pocket (OOP) is ~36%, and medical insurance coverage is <8% in the region (WHO, 2011, 2018b). Some countries, such as Cameroon, Equatorial Guinea and Nigeria, even had OOP health expenditures exceeding 70% of their total health expenditure in 2019 (World Bank, 2022). The high OOP burden from NCD care in SSA is due to limited financial risk protection mechanisms such as health insurance and social support systems, high costs and prolonged treatment of NCDs such as cancer, mental health problems, renal diseases and their resulting disability. Moreover, with a high number of poorer households in SSA spending a higher proportion of their income on NCDs, a small OOP will likely predispose them to financial hardships. For example, in Nigeria, CHE due to NCDs accounts for 81.7% of the poorest households and 8% of the richest households (Odunyemi *et al*., 2023). These also account for the disparity in OOP expenditures observed between countries.

Given the increasing and unique risks of the financial impact of OOP spending on NCDs in SSA, synthesized evidence is paramount for effectively monitoring the progress of the attainment of the health-related SDGs. A synthesis of the extant literature will assist in locating gaps in evidence and provide an evidence-based pedestal for targeted policy implementations (Juma *et al*., 2019; Tesema *et al*., 2020; Rahman *et al*., 2022b). However, there is a shortage of such studies in LMICs, particularly in SSA (Muka *et al*., 2015; Kazibwe *et al*., 2021). There are only a handful of systematic or scoping reviews on the economic impacts of NCDs in LMICs (Kankeu *et al*., 2013; Jan *et al*., 2018; Rijal *et al*., 2018; Kazibwe *et al*., 2021). Most of these studies focused only on the distribution of NCD health costs (direct or indirect) without indicators of household financial burden (Kankeu *et al*., 2013; Gheorghe *et al*., 2018; Moucheraud *et al*., 2019; Kazibwe *et al*., 2021; Gnugesser *et al*., 2022; Mattap *et al*., 2022; Mutyambizi-Mafunda *et al*., 2023) and/or were restricted to one or two NCDs (Gheorghe *et al*., 2018; Moucheraud *et al*., 2019; Gnugesser *et al*., 2022; Mattap *et al*., 2022).

One of the most recent review papers on the financial burden of OOP spending on NCDs in LMICs covered only five major NCDs and 1990–2016 period (Jan *et al*., 2018). Only three indicators of the financial burden of NCDs were surveyed: catastrophic health expenditure (CHE), impoverishment and coping strategies; studies on unmet needs for financial reasons (UNFR) and crowding-out effect were excluded (Jan *et al*., 2018). The focus of two other recent financial protection reviews was too broad, thereby excluding many NCD studies, particularly from SSA (Njagi *et al*., 2018; Rahman *et al*., 2022b). They also overlooked two or more of the crowding-out effects, distressed financing or UNFR, important indicators of the burden of OOP health spending. Other systematic reviews examined only household coping strategies (Rijal *et al*., 2018; Murphy *et al*., 2019) or CHE (Eze *et al.*, 2022a, 2022b). A recent umbrella review identified no relevant systematic review examining NCD-related OOP spending in low-income countries (LICs), most of which are in SSA (NCD Alliance, 2023). However, one targeted review was undertaken on economic evaluation studies on NCDs, mostly pharmacological interventions in SSA, which is entirely different in scope from our current review (Hollingworth *et al*., 2023). To the best of our knowledge, there is currently no broad-based evidence on the financial burden of NCDs in SSA. Therefore, we aim to fill these gaps by expanding our search beyond the five major NCDs, encompassing all indicators of the financial burden of OOP expenditures and evaluating their equity implications, scoping studies from SSA since the commencement of the SDGs. Our study has several objectives. First, we intend to summarize the findings and trends of studies that estimate the financial burdens of OOP spending on NCDs in SSA. Second, we seek to determine the extent and variation of the financial burden across different country income groups and the methodological approaches used to measure them. Finally, we evaluate the equity implications of these financial burden methods and estimates.

The novelty of our study lies in its exhaustive coverage, setting it apart from previous reviews. Unlike most prior studies that focused on the five major NCDs, our review encompasses a broader range of NCDs. Additionally, it provides a comprehensive coverage of the indicators of the financial burden of NCDs and their equity implication. Through its extensive analyses, our study offers valuable insights to guide NCD-related policies and practices in SSA.

## METHOD

### Conceptual framework and definition

**Fig. 1:**
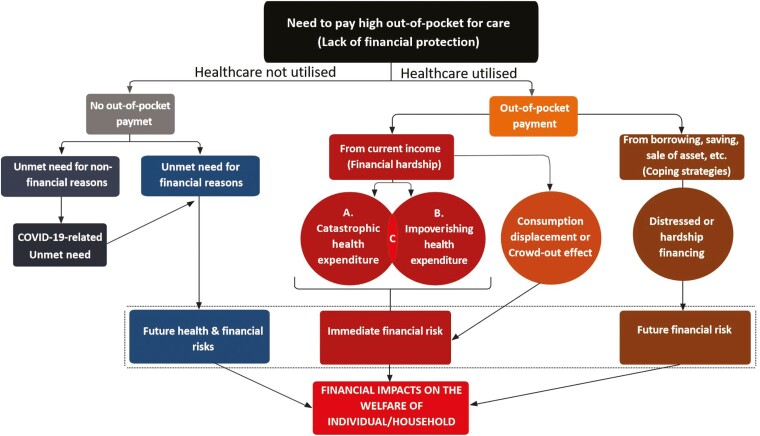
A conceptual framework of the financial burden of out-of-pocket (OOP) expenditures due to noncommunicable disease care. (A) Catastrophic health expenditure: OOP expenditure greater than a specified threshold of household income or consumption. (B) Impoverishing health expenditure: when the remaining household expenditure lies below the poverty line after OOP expenditure is removed either for those already below (further impoverished) or pushed below (impoverished) the chosen poverty line. (C) When OOP healthcare expenditure is both catastrophic and impoverishing for a household. Source: Authors’ own elaboration.

Figure 1 illustrates the pathways through which households with NCDs are at risk of the financial burden of OOP health payments (lack of **financial protection**). The nature of the burden depends on the household’s **capacity to pay (CTP)** and the source of OOP payments. When a household finances healthcare through current income or expenditure, it may experience **CHE, impoverishing health expenditure (IHE) or crowding-out effect (consumption displacement)**. CHE occurs when OOP expenditure exceeds a certain threshold of a household’s ability to pay, necessitating a significant cutback on essential consumption (Cylus *et al*., 2018; Nguyen *et al*., 2023; Owen, 2019). Depending on the definition of CTP, there are two broad approaches for estimating CHE. In the **budget share** approach, total household income or expenditure is used, and in the CTP approach, the household’s discretionary expenditure remaining after its basic needs have been met is used (Nguyen *et al*., 2023). The budget share method was adopted as the SDG indicator (SDG 3.8.2) to track the progress of universal health coverage (UHC) (WHO, 2023b). Two CTP methods have traditionally been used for CHE measurements (Odunyemi *et al*., 2023). These are the **actual food expenditure** and **partial normative food expenditure** methods. The actual food expenditure uses CTP derived from total household expenditure net of actual spending on food, whereas the partial normative food expenditure method deducts a standard amount representing subsistence food spending (Thomson *et al.*, 2016; Cylus *et al*., 2018; Thomson *et al*., 2019). However, the latter method avoids a negative CTP by using actual food expenditure when a household is below the subsistence level (Cylus *et al*., 2018).

These three traditional methods, particularly the budget share method, tend to underestimate CHE among poor people and overestimate it among rich people, thus making it difficult to identify poor households experiencing financial hardship (Cylus *et al*., 2018; Hsu *et al*., 2018). Therefore, in 2016, the WHO European Office developed a new and fully normative CTP method to overcome this problem (Thomson *et al.*, 2016). This new method includes food, rent and utilities for subsistence spending. Odunyemi *et al*. (2023) modified these subsistence spending components for use in Nigeria by adding clothing and removing electricity and heating. The resolution of the equity challenge using the WHO Europe method is important for pro-poor policy initiatives (Cylus *et al*., 2018). The equity implications of CHE estimations are vital to NCDs, which disproportionately affect the poor (WHO, 2023a). Unlike the partial normative food expenditure method, the WHO Europe method allows poor households to have a negative CTP (Thomson *et al*., 2019).

Before the emergence of the WHO Europe method, Ataguba (2012) developed a method called the **Ataguba method** to resolve the equity issue. This method employs a rank-dependent threshold (instead of the fixed threshold used in the methods) for CHE estimation. The choice of thresholds is arbitrary. For example, the budget share method commonly uses fixed thresholds of 10% and 25% (Grépin *et al*., 2020), the actual food spending method uses 25% and 40% and both the partial normative food expenditure and WHO Europe methods use 40% (Nguyen *et al*., 2023). Multiple fixed thresholds have often been used to investigate inequity from various perspectives (Nguyen *et al*., 2023). However, for equitable purposes, the Ataguba method, using a parameter of aversion to inequality, γ = s0.8, adopts variable thresholds that allow poor households to face lower thresholds than rich households. The Ataguba method is useful where inequalities are high, such as SSA (Mutyambizi *et al*., 2019).

The CHE calculated from the above methods is called the **catastrophic headcount ratio**. Beyond the headcount ratio, various indices have been used to illustrate the intensity of CHE. **Catastrophic overshoot** provides the degree to which OOP spending, as a proportion of income or expenditure, exceeds **(positive overshoot**) or drops below (**negative overshoot**) the CHE threshold. The **mean positive overshoot** is the ratio of positive overshoot to the headcount ratio. While the positive overshoot and mean positive gap (overshoot) provide insight into the extent of the burden of OOP expenditure on healthcare beyond the threshold, a negative overshoot suggests a lower CHE risk for a household (Piroozi *et al*., 2020). It has been shown that the intensity of CHE responds better to policy changes than the headcount ratio (Koo and Jung, 2022).

For equity analysis of CHE, households are usually ranked from the poorest to the richest (or quintile) according to their socioeconomic status (McIntyre and Ataguba, 2010). For a robust analysis of equity, **progressivity analysis** or **financial incidence analysis** is preferred (Ataguba *et al*., 2018). Three interrelated indicators were commonly used for this analysis: the **Gini index or coefficient (and Lorenz curve)**, **concentration index (and curve)** and **Kakwani index**.

IHE or, simply, impoverishment occurs when a household falls below the poverty line after OOP expenditure (**impoverished**) (see Figure 1). If the household was previously below the poverty line and pushed further down, it is said to be **further impoverished** (Thomson *et al*., 2019). The WHO Europe method identified three other categories of households above the poverty line: those without OOP expenditures (non-spenders) and those (**not) at risk of impoverishment** after OOP payments (Thomson *et al.*, 2016, 2019). Below the poverty line, a household’s basic standard of living is seriously jeopardized (Saksena *et al*., 2014). For households with CHE, there is a high probability of being plunged down the poverty line by OOP spending (Nguyen and Nguyen, 2020; Panikkassery, 2020; Wei *et al*., 2021). An extremely poor household may experience CHE and IHE concurrently (labelled C in Figure 1). The estimate of this overlap is ~9% of the population in LMICs (WHO, 2023b). A household with CHE and/or IHE is said to experience **financial hardship** (Thomson *et al*., 2019).

**The crowding-out effect** is the extent to which household consumption is sacrificed for OOP expenditure (Pal, 2012; Panikkassery, 2020; Batbold *et al*., 2021) (Figure 1). Initially, households protect essential household consumption items such as food, clothing, rent and education, conserving them in lieu of non-essential consumption (Pal, 2012; Alam and Mahal, 2014). With higher OOP health expenditures, poor households are also forced to reduce consumption of essential items (Kumara and Samaratunge, 2017; Molla and Chi, 2018). Among the essential consumption items, food is usually prioritized (Pal, 2012; Panikkassery, 2020). Therefore, when food expenditure is affected, it indicates extreme living conditions, irrespective of whether such household’s consumption is above or below the CHE threshold or poverty line.

To protect essential consumption, households may devise other means apart from their current income to pay for healthcare. These are known as **coping strategies** (see Figure 1). Common coping strategies include dissaving, borrowing and disposing of assets. When borrowing or sale of assets is used, it is called **distressed financing** because it is an extreme coping strategy (Kruk *et al*., 2009). In some cases, children are withdrawn from school or engaged in child labour to pay for medical expenses (Kankeu *et al*., 2013; Mirelman *et al*., 2019; Murphy *et al*., 2019). While coping strategies may help households maintain consumption in the short run, they may increase the risk of future impoverishment and indebtedness (Chhay and Rahut, 2022) (Figure 1). Households with NCDs are more likely to use coping strategies because of high costs and prolonged treatment duration (Jan *et al*., 2018; Murphy *et al*., 2019; Sheikh *et al*., 2022). A study can exaggerate the risk of household consumption and, thus, CHE and IHE or overlook the long-term burden of OOP payments for chronic diseases, such as NCDs, if these coping strategies are not considered (Flores *et al*., 2008; Wagstaff, 2019).

The exorbitant cost of NCD care can cause people to delay or forgo care because of unaffordable OOP payments (Rahman *et al*., 2022a; Lombe *et al*., 2023). This is called the **unmet need for financial reasons (UNFR)** (Thomson *et al.*, 2016) (see Figure 1). UNFR often has devastating and prolonged consequences for the health outcomes of people with NCDs (Hsu *et al*., 2018; Gabani and Guinness, 2019; Petrovic *et al*., 2021). Since CHE and IHE only capture the financial burden among people who utilize care, they would give falsely low estimates where, as common with NCDs, there is a large UNFR (Thomson *et al.*, 2016; Murphy *et al*., 2020). Therefore, to better interpret CHE and IHE, the inclusion of the UNFR is recommended (Thomson *et al*., 2019). Although the COVID-19 pandemic was a nonfinancial cause of unmet needs, financial reasons topped the reason for widespread unmet needs during the pandemic and should be considered separately (Chang *et al*., 2021) (see Figure 1).

Although CHE is the only indicator of financial protection in the SDGs and is usually examined in the literature with IHE, there is an emerging consensus on the need for a composite indicator to capture the multifaceted dimensions of financial protection analyses (Moreno-Serra *et al*., 2011; Hsu *et al*., 2018).

### Eligibility criteria

This review was conducted based on predefined inclusion and exclusion criteria. These eligibility criteria followed the participant, concepts and context (PCC) framework and included other study characteristics, such as study design and publication type.

#### Participants

We included individuals of all ages with an NCD. Participants were excluded if they had infectious, maternal, neonatal, nutritional or metabolic diseases; primary NCD risk factors other than hypertension or diabetes (e.g. smoking, alcohol consumption, overweight, obesity, dyslipidaemia, hypercholesterolaemia, atherosclerosis or air pollution); preventive care for NCDs (e.g. diabetes, hypertension or precancerous screening) or studies that combined NCDs with other chronic infectious diseases or studies conducted in nonhuman subjects.

#### Concepts (indicators of financial burden)

The concepts included any original study that used primary or secondary data to produce economic estimates (quantitative analysis) of the burden of OOP expenditure (i.e. measuring the prevalence, distribution and trend of CHE, IHE, adoption of coping strategies, crowding-out effect and UNFR). The following concepts were excluded: studies without an OOP expenditure/cost component; studies on indirect costs (e.g. transportation cost and productivity loss); studies on clinical or cost-effective analysis or economic evaluation of drugs or treatment; all non-empirical studies and those on nonfinancial barriers to accessing care or cancer financial toxicity (subjective financial impact) (Witte *et al*., 2019).

#### Context

Studies from any SSA country, as defined by the World Bank, were included (World Bank, 2021).

#### Study characteristics

We included case-control studies, cross-sectional studies, cohort studies, mixed-method studies, peer-reviewed and grey literature (e.g. pre-print articles, dissertations, working papers and reports) published in English between 1 January 2015 and 18 August 2023 (both inclusive). However, the following publication types were excluded: case reports, case series, literature reviews (e.g. systematic reviews, scoping reviews, and narrative reviews), study protocols, policy papers, newspaper articles, editorials and letters to editors, commentaries and opinion pieces/perspectives and corrections and retracted publications.

### Search strategies

We followed the PCC framework for scoping reviews to develop our search strategy and search strings (Joanna Briggs Institute (JBI), 2020). The details of the search strategies and search strings are contained in Appendix I and Supplementary Table S1.

### Screening and selection

**Fig. 2:**
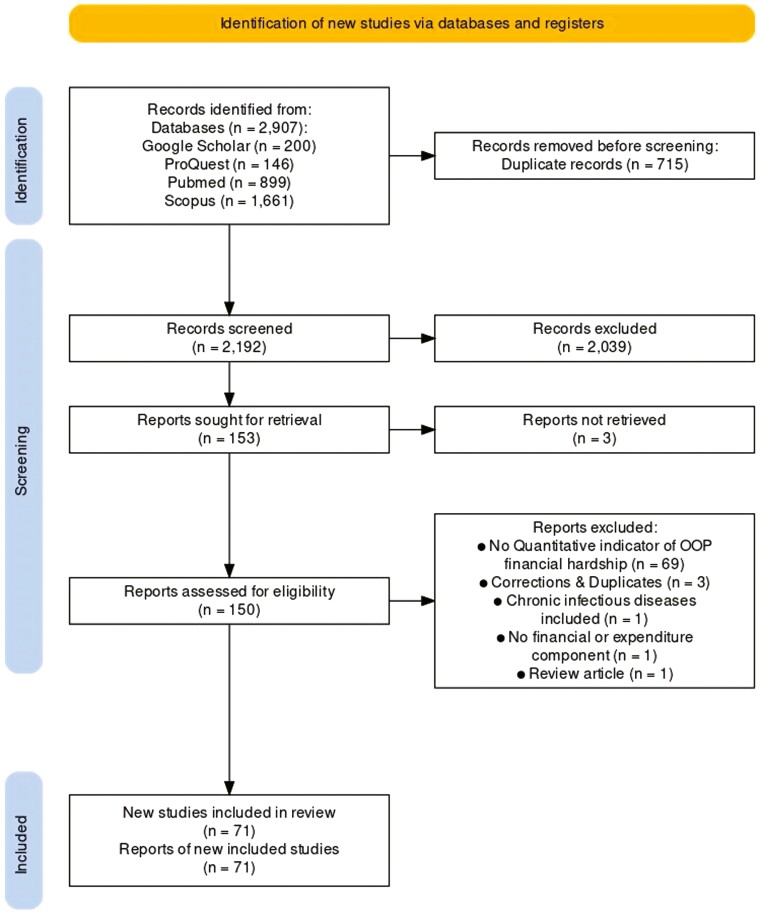
PRISMA-ScR flow chart of the study selection.

We followed the updated Preferred Reporting Items for Systematic Reviews and Meta-Analyses Extension for Scoping Reviews (PRISMA-ScR) guidelines during the study selection process (Figure 2) (Peters *et al*., 2021). The retrieved studies from all the databases were uploaded to the Nested-Knowledge AutoLit platform, which automated study deduplication. The nonduplicate records were subsequently screened in two consecutive stages following predefined eligibility criteria. Title and abstract screenings were independently and systematically conducted by AO and reviewed by MTI (Supplementary Figure S1). Subsequently, an initial full-text screening was undertaken by AO. The tentatively included studies were reviewed by MTI to determine whether they should be included, and discrepancies were resolved by consultation with the senior author (K.A.) and consensus among the authors.

### Data extraction and synthesis

Following our research question, the study characteristics (authors, publication year, study design, survey type and setting) and outcome data (based on our conceptual framework) from each study were recorded in a data extraction form, which was initially piloted on a few studies. The data were extracted by A.O. and verified by M.T.I. and K.A.

We surveyed the quantity and trend of studies on the financial burdens of NCDs in 48 countries in SSA and their economic groups based on the 2022 World Bank Classification of Countries into LICs, lower-middle-income countries (LwMICs) and upper-middle-income countries (UMICs) (World Bank, 2021). We summarized the characteristics of those studies and the indicators employed in measuring the financial burden of NCDs. We then analysed the distribution of these indicators, their methodological approaches and the depth of analysis.

## RESULTS

### Overview of the included studies

A total of 71 studies, consisting of 70 peer-reviewed articles, were included in this analysis. Sixty-eight of them were purely quantitative, including 55 cross-sectional (77.5%) and 13 (18.3%) cohort studies, representing 95.8% of all the studies (Table 2). The remaining three studies (4.2%) were mixed. Three studies employed a comparative design and included a control group (Mwai and Muriithi, 2016; Murphy *et al*., 2020; Kitole *et al*., 2022). Health facility-based studies dominated the overall survey type, comprising 73.2% of the facility-only studies and 1.4% of the mixed surveys. Only 12.7% (9) of the studies utilized nationally representative household survey data. Surprisingly, none of the cohort studies examined the longitudinal trends of the indicators. There was a significant decrease in the number of publications between 2017 and 2019, after which there was an upward trend, peaking in 2021, followed by another downward trend (Supplementary Figure S2). The annual publication rate is lower for countries in the UMIC compared with LwMICs and LICs.

### The geographical distribution of studies

**Fig. 3:**
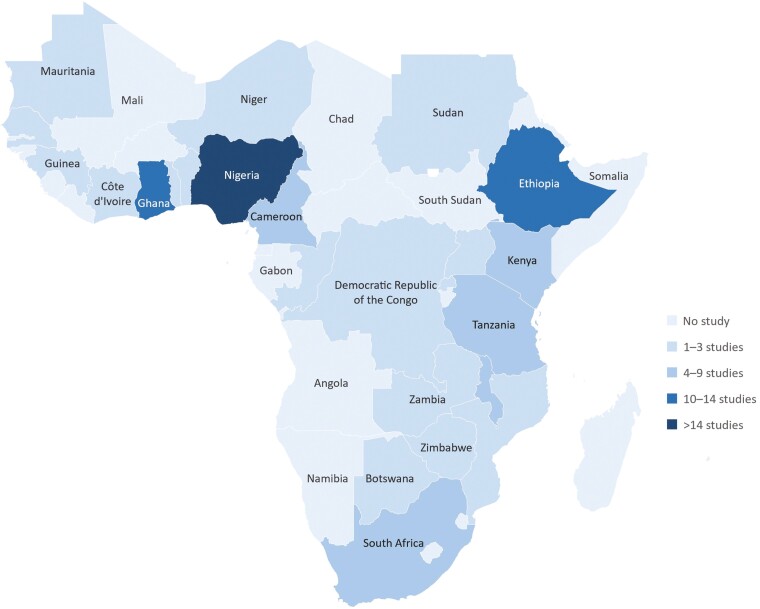
The geographical distribution of the included studies. *Note:* For multi-country studies, every country is represented in the figure so that the total number of studies depicted in the figure typically exceeds the number of included studies in the review.

Most of the included studies originated from a single country, and only 9.9% were from multiple countries (Table 2). Approximately 50% of the countries in SSA had no studies at all (Figure 3). The West African subregion had the highest number of studies (46%), with >14 studies (~20%) originating from Nigeria. Ghana (also in West Africa) and Ethiopia (in Eastern Africa) had 10–14 studies each. Among the three, only Ghana was included in the multi-country studies.

### Overview of noncommunicable diseases studied

Four major NCDs [cancers (39.4%), CVD (36.6%), diabetes (22.5%) and chronic respiratory diseases (15.5%)] dominated the studies (Table 1). Apart from sense organ disorders (12.7%), the prevalence of other NCDs was <10%.

**Table 1:** Characteristics of the included studies, the types of indicators and the types of noncommunicable diseases examined

Study	Study design	Survey type	Country (region, economic group)	Indicator of financial burden				NCD category
				CHE	IHE	CS	UNFR	
(Odunyemi *et al*., 2023)	Observational: cross-sectional	Household survey (national)/22,200 households	Nigeria (WA, LwMIC)	✓	✓			Cancers; CVD; chronic respiratory diseases; diabetes; mental disorders; nervous system diseases; chronic renal disorders; haematologic diseases; sense organs disorder; gastrointestinal diseases; musculoskeletal disorders; other NCDs
(Seifu *et al.*, 2024)	Observational: cross-sectional (descriptive)	Health facility-based survey (multicentre)/318 individuals	Ethiopia (EA, LIC)				✓	Chronic renal disorders
(Kambhampati *et al.*, 2023)	Observational: cross-sectional	Health facility-based survey (multicentre)/40 individual (women)	Botswana (SA, UMIC); Zimbabwe (SA, LwMIC)				✓	Cancers (cervical cancer)
(Ocran Mattila *et al*., 2023)	Observational: cross-sectional	Health facility-based survey (multicentre)/29 facilities	Ghana (WA, LwMIC)				✓	Cancers
(Adanu *et al.*, 2022)	Observational: cross-sectional	Health facility-based (single centre)/74 individuals	Ghana (WA, LwMIC)			✓		Cancers (breast cancer)
(Adeniji and Obembe, 2023)^§^	Observational: cross-sectional	Household survey (national)/9333 individuals	Ghana (WA, LwMIC); South Africa (SA, UMIC)	✓				CVD
(Degefa *et al*., 2023)	Observational: cross-sectional	Health facility-based survey (single centre)/325 individuals	Ethiopia (EA, LIC)	✓				Chronic renal disorders; CVD; mental disorder; diabetes
(Bell *et al.*, 2022)	Observational: cross-sectional	Health facility-based survey (single centre)/100 participants	Ghana (WA, LwMIC)				✓	Cancers (cervical cancer)
(Kitole *et al*., 2022)^¶^	Observational: cross-sectional	National; household survey (national)/9465 households	Tanzania (EA, LwMIC)	✓	✓			Nonspecific
(Amarachukwu *et al*., 2022)	Observational: cross-sectional (descriptive)	Health facility-based survey (single centre)/ 149 participants	Nigeria (WA, LwMIC)	✓				Haematologic Diseases (sickle cell disease)
(Adeniji *et al.*, 2022)	Observational;:Cross-sectional (descriptive)	Health facility-based survey (multicentre)/ 744 participants	Nigeria (WA, LwMIC)	✓	✓			CVD
(Knapp *et al*., 2022)	Observational: longitudinal (prospective cohort)	Database analysis (breast cancer database)/ 635 participants	Nigeria (WA, LwMIC)	✓		✓	✓	Cancers (breast cancer)
(Endale *et al*., 2022)	Observational: cross-sectional	Health facility-based survey (single centre)/ 423 participants	Ethiopia (EA, LIC)			✓		Cancers (cervical cancer)
(Adeke *et al*., 2022)	Mixed: descriptive cross-sectional and focus group discussions	Health facility-based survey (multicentre)/ 45 facilities and 145 individuals	Nigeria (WA, LwMIC)				✓	CVD (hypertension)
(Jane Bates *et al.*, 2021)	Observational: longitudinal (prospective cohort)	Household survey (national)/ 150 households	Malawi (SA, LIC)	✓		✓		Cancers (advanced cancer)
(Reibold *et al.*, 2021)	Observational: longitudinal (prospective cohort)	Health facility-based survey (single centre)/101 participants	Ethiopia (EA, LIC)				✓	Cancers (breast cancer)
(Bizuneh *et al*., 2021)	Observational: cross-sectional	Health facility-based survey (multicentre)/ 209 participants	Ethiopia (EA, LIC)				✓	Sense Organs Disorders (cataract)
(Opara *et al*., 2021)	Observational: cross-sectional	Database analysis (National RHD Registry database)/ 87 participants	Uganda (EA, LIC)	✓		✓		CVD (rheumatic heart disease)
(Mattila *et al*., 2021)	Observational: cross-sectional	Database analysis (The South African Medicines Price Registry)/1 database	South Africa (SA, UMIC)				✓	Cancers (breast, prostate & colorectal)
(Mensah *et al*., 2021)	Observational: cross-sectional	Health facility-based survey (multicentre)/ 6 pharmacies	Ghana (WA, LwMIC)				✓	Cancers (childhood cancers)
(Akpan *et al*., 2020)	Observational: longitudinal (retrospective cohort)	Health facility-based survey (single centre)/ 613 participants	Nigeria (WA, LwMIC)				✓	Chronic Renal Disorders
(Baatiema *et al.*, 2021)	Observational: cross-sectional	Health facility-based survey (multicentre)/ 136 participants	Ghana (WA, LwMIC)				✓	CVD (stroke)
(Hagos *et al*., 2020)	Observational: cross-sectional	Health facility-based survey (multicentre)/ 464 participants	Ethiopia(EA, LIC)			✓		Cancers (cervical, colorectal, leukemia; lung, gastro-esophage, live, ovarian, rectal, testicular, non-Hodgkins lymphoma)
(Tadesse and Beyene, 2020)	Observational: cross-sectional	Health facility-based survey (single centre)/ 94 participants	Ethiopia (EA, LIC)				✓	Chronic Respiratory Diseases (asthma)
(Kasahun *et al*., 2020)	Observational: cross-sectional: (hospital-based)	Health facility survey (multicentre)/ 404 participants	Ethiopia (EA, LIC)	✓		✓		Cancers
(Murphy *et al*., 2020)^¶^^§^	Observational: longitudinal (Prospective cohort)	Household survey (national)/ 70346 households	Tanzania (EA, LwMIC); Zimbabwe (SA, LwMIC); South Africa (SA, UMIC)	✓	✓	✓	✓	Chronic Renal Disorders; Chronic Respiratory Diseases (asthma); Cancers; Diabetes
(Mamo *et al.*, 2019)	Observational: cross-sectional	Health facility-based (multicenter)/ 268 (case) and 211(control) participants	Ethiopia (EA, LIC)				✓	Neurological diseases (epilepsy); CVD (hypertension)
(Sharma *et al.*, 2019)	Observational: longitudinal: (prospective cohort)	Database analysis (prospective cancer database)/ 300 participants	Nigeria (WA, LwMIC)				✓	Cancers
(Macquart de Terline *et al.*, 2019)	Observational: cross-sectional	Health facility-based survey (multicentre)/ 2198 participants	Cameroon (CA, LwMIC); Gabon (CA, UMIC); DRC (CA, LIC); Congo Brazzaville (CA, LwMIC); Mozambique (SA, LIC); Togo (WA, LIC); Senegal (WA, LwMIC); Niger (WA, LIC); Mauritania (WA, LwMIC); Guinea (WA, LIC); Côte d’Ivoire (WA, LwMIC); Benin (WA, LwMIC)				✓	CVD (hypertension)
(Oyando *et al*., 2019)	Observational: cross-sectional	Health facility-based survey (multicenter)/ 212 participants	Kenya (EA, LwMIC)	✓		✓		CVD (hypertension)
(Ramlucken and Sibiya, 2018)	Observational: cross-sectional (descriptive)	Health facility-based survey (multicenter)/ 182 participant	South Africa (SA, UMIC)				✓	Mental Disorders (psychotic disorder), depression, anxiety
(Tolla *et al*., 2017)	Observational: cross-sectional	Health facility-based survey (multicenter)/ 589 participants	Ethiopia (EA, LIC)	✓		✓		CVD (ischaemic heart disease (IHD), stroke, hypertension and dyslipidaemia)
(Okediji *et al*., 2017)	Observational: cross-sectional	Health facility-based survey (multicenter)/ 443 participants	Nigeria (WA, LwMIC)	✓		✓	✓	Chronic Respiratory Diseases (asthma), Mental Disorders; Musculoskeletal disorders; Gastrointestinal Diseases; Chronic Renal Disorders; Diabetes; Cancers
(Worku *et al.*, 2017)	Observational: cross-sectional	Health facility-based survey (single center)/ 423 participants	Ethiopia (EA, LIC)				✓	Cancers (breast, colorectal, cervical cancers, lymphoma, lung, leukemia, kidney and prostate cancers)
(Awad *et al.*, 2017)	Observational: cross-sectional	Health facility survey (multicentre)/ 433 participants	Sudan (EA, LIC)				✓	CVD
(Wang *et al.*, 2016)	Observational: cross-sectional	Household survey (local)/ 1199 households	Malawi (SA, LIC)	✓	✓			Sense Organs Disorders; Gastrointestinal Diseases; Musculoskeletal disorders; Mental Disorders; Chronic Respiratory Diseases; CVD
(Goeppel *et al*., 2016)^§^	Observational: cross-sectional	Household survey (national)/ 16631 participants	South Africa (SA, UMIC); Ghana (WA, LwMIC)	✓				Musculoskeletal disorders arthritis; Mental Disorders (depression); Chronic Respiratory Diseases (asthma); Diabetes; CVD (hypertension, stroke, angina)
(Kanyamuhunga *et al*., 2015)	Observational; Longitudinal (prospective descriptive)	Health facility-based survey (single centre)/ 25 participants	Rwanda (EA, LIC)				✓	Cancers (childhood cancer)
(Abdull *et al*., 2015)	Observational: cross-sectional	Health facility survey (single centre)/ 131 participants	Nigeria (WA, LwMIC)				✓	Sense Organs Disorders (glaucoma)
(Ibrahim *et al.*, 2015)	Observational: cross-sectional	Health facility survey (single centre)/ 104 participants	Nigeria (WA, LwMIC)			✓		Sense Organs Disorders (cataract)
(Njuguna *et al.*, 2015)	Observational: cross-sectional	Health facility survey (single centre)/ 115 participants	Kenya (EA, LwMIC)				✓	Cancers (childhood cancer)
(Griffiths *et al*., 2015)	Observational: cross-sectional	Health facility survey (single centre)/ 90 participants	Zambia (SA, LwMIC)			✓		Sense Organs Disorders (refractive error and cataract)
(Teshome *et al*., 2021)	Observational: cross-sectional	Health facility survey (single centre)/ 196 participants	Ethiopia (EA, LIC)				✓	Cancers (breast cancer)
(Janssens *et al.*, 2016)	Observational: cross-sectional	Household survey (local)/ 1,450 households	Nigeria (WA, LwMIC)	✓				Haematologic Diseases (sickele cell diseases); Neurological (epilepsy) Diseases; Musculoskeletal disorders (low back pain, arthritis, spinal disorders, and trauma),; Diabetes; Chronic Respiratory (asthma) Diseases; CVD (myocardial attack or heart failure, hypertension)
(Mwai and Muriithi, 2016)^¶^	Observational: cross-sectional	Household survey (national)/ 8,844 household	Kenya (EA, LwMIC)	✓	✓			Nonspecific
(Ipinnimo and Durowade, 2022)	Observational: cross-sectional	Health facility-based survey (multicentre)/ 360 participants	Nigeria (WA, LwMIC)	✓	✓			Diabetes; CVD (hypertension)
(Subramanian *et al*., 2018)	Observational: cross-sectional	Household survey(national)/ 33,675 households	Kenya (EA, LwMIC)	✓			✓	Diabetes; Chronic Respiratory Diseases; CVD; Cancers;
Bates, (2021)*	Mixed: Photovoice (a community-based visual participatory action research method) and cross-sectional	Health facility survey (single centre)/ 150 participants	Malawi (SA, LIC)	✓				Cancers (Kaposi’s sarcoma, cervical cancer, oesophageal cancer, or hepatocellular carcinoma)
(Oyando *et al*., 2023)	Observational: longitudinal (prospective cohort)	Household survey (local)/ 888 households	Kenya (EA, LwMIC)	✓				Diabetes; CVD (hypertension)
(Ambrose *et al.*, 2023)	Observational: cross-sectional	Health facility-based survey (single centre)/ 87 participants	Tanzania (EA, LwMIC)				✓	Haematologic Diseases (sickle cell disease
(Haakenstad *et al.*, 2022)^§^	Observational: cross-sectional	Household survey (national) 44,089 participants	South Africa (SA, UMIC); Ghana (WA, LwMIC)	✓				Diabetes; Musculoskeletal disorders (chronic pain in joints/arthritis); Gastrointestinal Diseases; Mental Disorders (depression or anxiety); CVD (heart problems, hypertension, stroke); Cancers
(Beaudoin *et al.*, 2022)	Mixed: qualitative ethnographic and cross-sectional descriptive	Health facility-based survey (multicentre)/ 33 participants	Senegal (WA, LwMIC)				✓	Cancers (head and neck cancer)
(Chianumba *et al*., 2022)	Observational: cross-sectional	Health facility-based (multicentre)/ 378 participants	Nigeria (WA, LwMIC)				✓	Haematologic Diseases (sickle cell disease)
(Chagaluka *et al.*, 2021)	Observational: longitudinal (prospective cohort)	Health facility survey (multicentre)/ 252 participants	Zimbabwe (SA, LwMIC); Ghana (WA, LwMIC); Cameroon (CA, LwMIC); Kenya (EA, LwMIC); Malawi (SA, LIC)				✓	Cancers
(Ughasoro *et al*., 2021)	Observational: longitudinal (prospective cohort)	Health facility-based survey (single centre)/ 200 participants	Nigeria (WA, LwMIC)	✓				Chronic Respiratory Diseases (asthma)
(Ibukun and Adebayo, 2021)	Observational: cross-sectional	Health facility-based survey (multicentre)/ 1,320 participants	Nigeria (WA, LwMIC)	✓	✓			Diabetes; Chronic Respiratory Diseases; CVD; Cancers
(Tsega *et al*., 2021)	Observational: cross-sectional	Health facility-based survey (multicentre)/ 422 participants	Ethiopia (EA, LIC)	✓	✓	✓		Diabetes
(Dzudie *et al*., 2021)	Observational: longitudinal (retrospective cohort)	Database analysis (National pace-maker registry)/ 147 participants	Cameroon (CA, LwMIC)				✓	CVD
(Taryam *et al*., 2020)	Observational: cross-sectional	Household survey (local)/ 3,120 participants	Nigeria (WA, LwMIC)				✓	Sense Organs Disorders (blindness and visual impairment and cataract)
(Idris and Olaniyi, 2020)	Observational: cross-sectional	Health facility-based survey (multicentre)/ 255 participants	Nigeria (WA, LwMIC)	✓				CVD (hypertension)
(Tshivhase *et al*., 2020)	Observational: cross-sectional	Health facility-based survey (multicentre)/ 429 participants	South Africa (SA, UMIC)				✓	Sense Organs Disorders (glaucoma)
(Abegaz *et al*., 2020)	Observational: cross-sectional	Health facility-based survey (single centre)/ 307 participants	Ethiopia (EA, LIC)				✓	Chronic Respiratory Diseases (asthma)
(Pallangyo *et al*., 2020)	Observational: longitudinal (prospective cohort)	Health facility-based survey (single centre)/ 459 participants	Tanzania (EA, LwMIC)				✓	CVD (heart failure)
(Ingwu *et al.*, 2019)	Observational: cross-sectional (descriptive)	Health facility-based survey (single centre)/ 100 participants	Nigeria (WA, LwMIC)				✓	Cancers (breast cancer)
(Mutyambizi *et al*., 2019)	Observational: cross-sectional	Health facility-based survey (multicentre)/ 396 participants	South Africa (SA, UMIC)	✓	✓			Diabetes
(Tchounzou *et al.*, 2019)	Observational: longitudinal (prospective cohort)	Database analysis (in-hospital gynecologic cancer registry)/ 126 participants	Cameroon (CA, LwMIC)				✓	Cancers (cervical cancer)
(Nketia-Kyere *et al*., 2017)	Observational: cross-sectional	Health facility survey (single centre)/ 207 participants	Ghana (WA, LwMIC)				✓	CVD (stroke)
(Mboni *et al*., 2016)	Observational: cross-sectional	Health facility survey (single centre)/70 participants	Zambia (SA, LwMIC)				✓	Sense Organs Disorders (cataract)
(Okoronkwo *et al*., 2016)	Observational: Cross-sectional	Health facility-based survey (single centre)/292 participants	Nigeria (WA, LwMIC)			✓		Diabetes
(Okoronkwo *et al.*, 2015)	Observational: cross-sectional	Health facility-based survey (single centre)/308 participants	Nigeria (WA, LwMIC)	✓				Diabetes
(Jackson *et al.*, 2015)	Observational: cross-sectional	Health facility survey (multicentre)/360 participants	Nigeria (WA, LwMIC)				✓	Diabetes
**Summary**	Cross-sectional: 55 (77.5%) Longitudinal (cohort): 13 (18.3%) Mixed (cross-sectional and qualitative): 3 (4.2%)	Household survey (national): 8 (11.3%) Household survey (local): 4 (5.6%) Health facility-based survey (single centre): 25 (35.2%) Health facility-based survey (multicentre): 27 (38.0%) Database analysis: 6 (8.5%) Mixed (Household (national) and health facility-based): 1 (1.4%)	Country coverage Single-country: 64 (90.1%) Multicounty: 7 (9.9%) Subregion CA: 4 (5.6%) EA: 27 (38.0%) SA: 16 (22.5%) WA: 33 (46.5%) Economic group LIC: 22 (31.0%) LwMIC: 47 (66.2%) UMIC:10 (14.1%)	29 (40.8%)	10 (14.1%)	15 (21.1%)	40 (56.3%%)	Nonspecific: 2 (2.8%) Cancers: 28 (39.4%) CVD: 26 (36.6%) Chronic respiratory diseases: 11 (15.5%) Diabetes: 16 (22.5%) Mental disorders: 7 (9.9%) Nervous system diseases: 3 (4.2%) Chronic renal disorders: 6 (8.5%) Haematologic diseases: 5 (7.0%) Sense organs disorders: 9 (12.7%) Gastrointestinal diseases: 4 (5.6%) Musculoskeletal disorders: 6 (8.5%) Other NCDs: 2 (2.8%)

Notes: *LIC*, low-income country; *LwMIC*, lower-middle-income country; *UMIC*, upper-middle-income country; *CE*, Central Africa; *EA*, Eastern Africa; *SA*, Southern Africa; *WA*, Western Africa, depicting the economic group by World Bank, 2022 classification of countries by income and region (https://datahelpdesk.worldbank.org/knowledgebase/articles/906519-world-bank-country-and-lending-groups).

*CHE*, catastrophic health expenditure; *IHE*, impoverishing health expenditure; *CS*, coping strategies; *UNFR*, unmet need for financial reason; *CVD*, cardiovascular diseases.

Apart from study-specific characteristics: ‘Study Design’, ‘Survey Type’ and ‘Country Coverage’, the sum of the percentages for the different items under the same column exceeds 100% because an item may appear in more than one study.

^§^These multi-country studies contain other countries outside the sub-Saharan region.

^*^Grey literature, representing 1.4% of all included studies.

^¶^Studies that include a control group.

### Overview of the indicators of financial burden of out-of-pocket expenditure examined

There were no studies on the crowding-out effect of OOP expenditure. Although UNFR rarely co-occurring with other indicators, UNFR was the most commonly used single indicator, representing 42.5% of all indicators and appearing in 56.3% of the studies (Tables 1 and 2). This was closely followed by CHE, which occurred in 40.8% of the studies, representing 30.8% of all the indicators. CHE was examined alone (11.7%) or with one or more of the other three indicators (67%). IHE was never used without CHE and was the most commonly occurring indicator with CHE in LwMIC. Coping strategies were the most commonly used indicator of CHE in LIC. Apart from IHE, most indicators were used alone rather than in combination with others in 58.9% of instances, and they were seldom combined in three (3.2%) or four (1.1%).

**Table 2:** Distribution of the four indicators used in the studies

Indicators	LIC	LwMIC	HIC	Multi-country	Total
CHE only	2 (7.4)	6 (11.5)	0(0.0)	3 (30.0)	11 (11.7)
Impoverishment only	0 (0.0)	0 (0.0)	0 (0.0)	0 (0.0)	0 (0.0)
DF only	2 (7.4)	4 (7.7)	0 (0.0)	0 (0.0)	6 (6.4)
UNFR only	10 (37.0)	20 (38.5)	3 (60.0)	3 (30.0)	36 (38.3)
Any one indicator	14 (51.8)	30 (57.7)	3 (60.0)	6 (60.0)	53 (58.9)
CHE and impoverishment	1 (3.7)	6 (11.5)	1 (20.0)	0 (0.0)	8 (8.5)
CHE and DF	4 (14.8)	1 (1.9)	0 (0.0)	0 (0.0)	5 (53.2)
CHE and UNFR	0 (0.0)	1 (1.9)	0 (0.0)	0 (0.0)	1 (1.1)
Any two indicators	5 (18.5)	8 (15.4)	1 (20.0)	0 (0.0)	14 (14.9)
CHE, impoverishment and CS	1 (3.7)	0 (0.0)	1 (20.0)	0 (0.0)	2 (2.1)
CHE, impoverishment and UNFR	0 (0.0)	0 (0.0)	0 (0.0)	0 (0.0)	0 (0.0)
CHE, CS and UNFR	0 (0.0)	2 (3.8)	0 (0.0)	0 (0.0)	2 (2.1)
Any three indicators	1 (3.7)	2 (3.8)	0 (0.0)	0 (0.0)	3(3.2)
All four indicators (CHE, impoverishment, CS and UNFR)	0 (0.0)	0 (0.0)	0 (0.0)	1 (10.0)	1 (1.1)
CHE (total)	8 (29.6)	16 (30.8)	1 (20.0)	4 (40.0)	29 (30.8)
Impoverishment (total)	2 (7.4)	6 (11.5)	1 (20.0)	1 (10.0)	10 (10.6)
CS (total)	7 (25.9)	7 (13.5)	0 (0.0)	1 (10.0)	15 (16.0)
UNFR (total)	10 (37.0)	23 (44.2)	3 (60.0)	4 (40.0)	40 (42.5)
Total (All)	27 (100)	52 (100)	5 (100)	10 (100)	94 (100)

*CHE*, catastrophic health expenditure; *CS*, coping strategies; *Impoverishment*, impoverishing health expenditure; *LIC*, low-income country; *LwMIC*, lower-middle-income country; *UMIC,* upper-middle-income country; *UNFR,* unmet need for financial reason.

### Measuring the financial burden of NCD care in sub-Saharan Africa

#### Catastrophic health expenditure estimates

The budget share and older CTP methods dominated the studies, especially for the LwMIC (Supplementary Table S2). The most frequently used single method was the budget share, representing 23.1% of all methods. After the budget share comes the partial normative food expenditure method (17.9%). As expected, newer and more equitable methods were sparingly used either singly or in combination with other methods (2.6%).

In most cases (84.8%), only the incidence of CHE (headcount ratio) was estimated, and the intensity of CHE was only measured in ~15% of cases. Although heterogeneity exists in the type of thresholds used (Supplementary Table S2), more studies employed a single fixed threshold (55.2%). Multiple thresholds were only employed 37.9% of the time, and the use of rank-dependent thresholds was uncommon.

Considering the analysis of equity or progressivity of CHE, the wealth quintile was the instrument of choice (90.5%) (Supplementary Table S2), and it was infrequently complemented by only the concentration index.

**Fig. 4:**
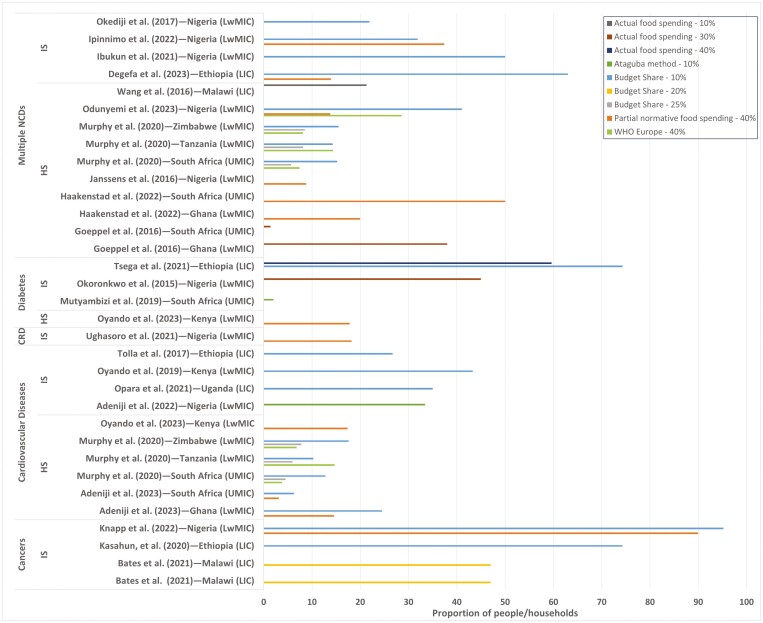
Proportion of individuals and households with catastrophic health expenditures (CHEs) on noncommunicable diseases (NCDs) at different thresholds and methods in sub-Saharan African countries and income groups. *Notes*: Two studies [Kitole *et al*. (2022) and Mwai and Muriithi (2016)] that only estimated the probability of incurring CHE (and not the actual values) were excluded. Where multiple thresholds were used for the same method, we chose the most commonly used methods (10% for budget share, 40% for actual food expenditure and partial normative food expenditure methods). For Ataguba rank-dependent (ƴ = 0.8), 10% and 40% threshold estimates were chosen when the denominator was income/total expenditure and nonfood expenditure approach, respectively. Ataguba with a fixed threshold of 10% (ƴ = 1) was renamed the ‘budget share’ since it yielded the same result. *LIC*, low-income country; *LwMIC*, lower-middle-income country; *UMIC*, upper-middle-income country; *WHO*, World Health Organization; *IS*, individual-level survey; *HS*, household-level survey; *HMD*, haematologic disease; *CRD*, chronic respiratory disease. The legend depicts the estimation method and threshold used.

There was variability in the CHE estimates depending on the country’s economic group, the type of NCDs, the type of survey and the method and threshold applied (Figure 4). Most estimations focused on three NCDs: cancer, cardiovascular disease (CVD) and diabetes. There were more studies on CVD than on other NCDs (Figure 4). There was only one study on haematologic (sickle cell) disease (Amarachukwu *et al*., 2022) and chronic respiratory diseases (Ughasoro *et al*., 2021). Heterogeneous findings on CHE were observed even between countries in the same economic group. Using the budget share method (at a 10% threshold), the CHE for NCDs ranged from 95.2% in LwMICs to 1.4% in UMICs. The highest CHE was for cancers in LwMIC (Knapp *et al*., 2022). Similarly, cancer incidence was high (74.3%) in LICs (Kasahun *et al*., 2020). As observed in Ethiopia and Uganda (Tolla *et al*., 2017; Opara *et al*., 2021; Tsega *et al*., 2021), CHE estimates for diabetes were higher than for CVD in LwMICs.

Most of the studies combined multiple NCDs with high CHE in LwMICs (individual-level CHE ranged from 31.9% to 50%) (Okediji *et al*., 2017; Ibukun and Adebayo, 2021; Ipinnimo and Durowade, 2022). The household-level CHE estimate was 41.04% (Odunyemi *et al*., 2023) in Nigeria compared to that in other LwMICs, such as Tanzania (14.3%) and Zimbabwe (15.5%) (Murphy *et al*., 2020). Generally, the CHE was lower for UMICs than for LwMICs. Except for one study (Degefa *et al*., 2023), CHE was unexpectedly lower in LICs than in LwMICs. Taking cancers as an example, the incidence of CHE in LIC (Ethiopia) (Kasahun *et al*., 2020) was approximately two-thirds that in LwMICs (Nigeria) (Knapp *et al*., 2022).

#### Disparity in catastrophic health expenditure estimates between the rich and poor

**Fig. 5:**
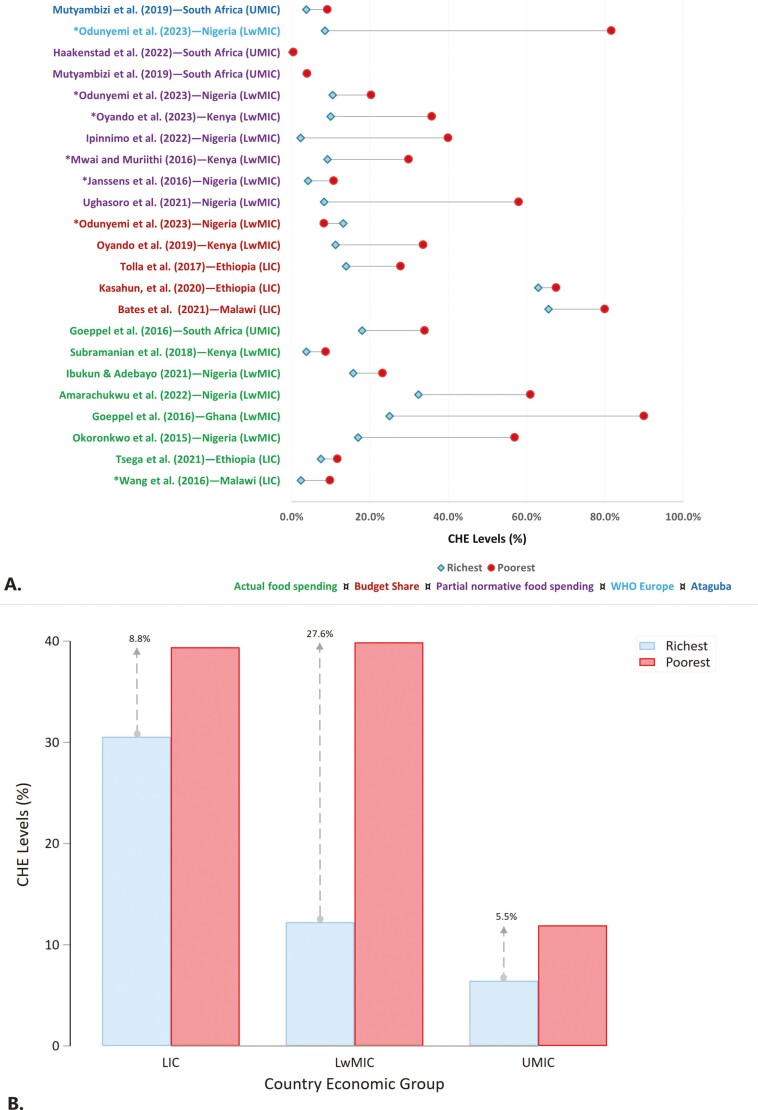
Disparity in catastrophic health expenditures (CHE) between the richest and poorest households with NCDs in sub-Saharan Africa. *Note:* Panel A shows the study-specific CHE for the poorest and richest households by estimation methods, thresholds and types of data (household or health facility) used. The colour of each study corresponds to that of the estimation method depicted in the legend. All methods used a 40% threshold except the budget share, which used 10%. *Studies that used household data (other studies used health facility data). Panel B shows the mean CHE for the poorest and richest households pooled together by country income group, irrespective of the estimation method, threshold or type of data (household or health facility) used. *LIC,* low-income country; *LwMIC,* lower-middle-income country; *UMIC,* upper-middle-income country.

Figure 5 illustrates the distribution of CHE between the richest and the poorest households in SSA. Most studies show >20% CHE, particularly for poor households (Figure 5A). Considering a similar estimation method, there is heterogeneity in these disparities based on country income levels and estimation methods. The poorest households bore a disproportionately high burden of CHE households in almost all countries (Figure 5B). UMICs show a relatively lower disparity between the richest and the poorest than LMICs and LICs. The disparities between the richest and the poorest are more pronounced in LwMICs than in LICs and UMICs, sometimes exceeding 50%, particularly in Nigeria and Ghana (Goeppel *et al*., 2016; Ughasoro *et al*., 2021; Odunyemi *et al*., 2023). While the disparity was lower for LICs than for LwMICs, the overall CHE percentages tended to be high across all income groups in LICs (Figure 5B).

Different methodologies yielded varying results regarding the disparities between the poorest and the richest households. As shown by studies using multiple methods (Mutyambizi *et al*., 2019; Odunyemi *et al*., 2023), compared with the WHO Europe and Ataguba methods, other methods consistently underestimated CHE among the poorest households within a country. The budget share method generally reported narrow gaps between the poorest and the richest households, and sometimes reversed the poor–rich disparity as in (Odunyemi *et al*., 2023). Additionally, Odunyemi *et al*. (2023), Oyando *et al*. (2023) and Mwai and Muriithi (2016), studies using household-level data, showed larger disparities between the poor and rich than facility-level data (Figure 5A).

#### Impoverishing health expenditures estimates

**Fig. 6:**
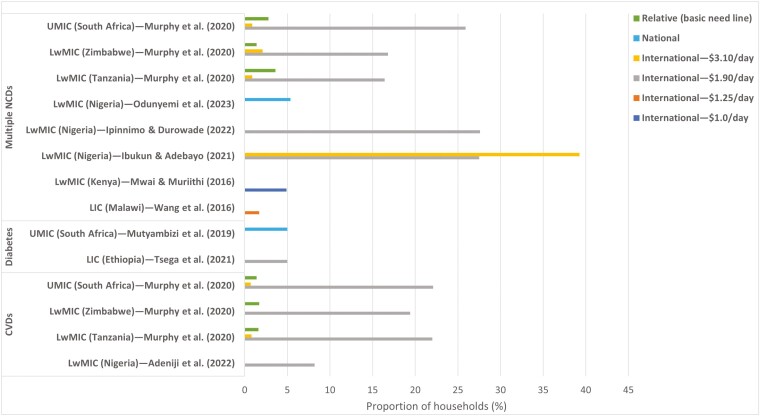
Proportion of households impoverished by health expenditures on noncommunicable diseases (NCDs) at different poverty levels in sub-Saharan African countries and economic groups. *Note:* A study [Kitole *et al*. (2022)] that only estimated the probability of impoverishment instead of actual values was excluded. *LIC,* low-income*; LwMIC,* lower-middle-income*; UMIC,* upper-middle-income; and *WHO,* World Health Organization.

Old traditional methods dominate (80%) the estimation methods for IHE, too (Supplementary Table S3). There was a lack of depth in the analyses, and only wealth quintiles were used for equity analysis. Most studies combined multiple NCDs, and only Diabetes and CVD were separately examined. There was also variability in the poverty lines used (Figure 6). Most studies used international poverty lines (particularly $1.90/day) instead of relative or national poverty lines.

The highest reported impoverishment (39.26%), using a $3.10/day poverty line, was found in a study from Nigeria (LwMIC) (Ibukun and Adebayo, 2021). With an international poverty line of $1.90/day, IHE from multiple NCDs for South Africa (UMIC) (25.9%) (Murphy *et al*., 2020) and Nigeria (27.6%) (Ibukun and Adebayo, 2021; Ipinnimo and Durowade, 2022) were very close. These figures are higher than those for Tanzania (16.4%) and Zimbabwe (16.8%), both of which are LwMICs with similar IHE. For CVD, using the $1.90/day and $3.10/day poverty lines from the same study (Murphy *et al*., 2020), UMICs (South Africa) unexpectedly had slightly greater impoverishment (22.1%) than LwMICs [Zimbabwe (19.4%) and Tanzania (22.0%)] did. The relative poverty line yielded completely opposite results, indicating that South Africa had lower IHE. There is not enough data to compare LICs with LwMICs or UMICs. For the same reason, disease-specific comparisons are difficult.

#### Coping strategy estimates

**Fig. 7:**
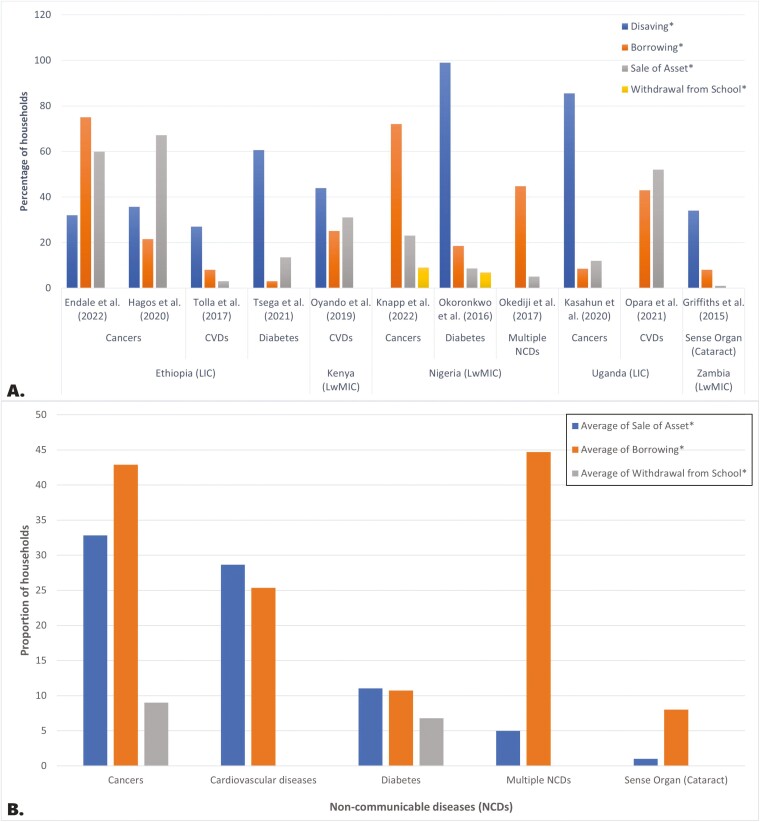
Proportion of households using different coping strategies (panel A) and distressed financing methods (panel B) in the sub-Saharan African countries by noncommunicable disease category. *Notes:* Studies without a specified proportion of people in a household using a coping strategy were excluded. ^*^Other uncommon coping strategies were not included in our analysis.

The four commonly used methods for coping strategies are dissaving, borrowing, selling assets and withdrawing children from school (Figure 7A). The latter method has rarely been examined, with only two studies from Nigeria including it (Okoronkwo *et al*., 2016; Knapp *et al*., 2022). As expected, these coping methods varied between countries and NCDs. Interestingly, there were no studies on coping strategies in UMICs. The studies included in the analysis were from two LICs (Ethiopia and Uganda) and three LwMICs (Nigeria, Kenya and Zambia) (Figure 7A). The study from Zambia examined sense organ diseases (Griffiths *et al*., 2015), and the remaining studies focused on cancer, CVD and diabetes.

Households with significant support from family, friends, government and nongovernmental organizations (NGOs) used more dissaving (Okoronkwo *et al*., 2016; Kasahun *et al*., 2020), and most households prefer dissaving over borrowing. Disavings and borrowing were more commonly used in LwMICs, while sales of assets were more common in LICs (Figure 7A). Extreme coping strategies or distressed financing, such as borrowing and asset sale, were more common for cancers and CVD (Oyando *et al*., 2019; Hagos *et al*., 2020; Opara *et al*., 2021; Endale *et al*., 2022; Knapp *et al*., 2022). The frequency of distressed coping strategies was greater for cancers than for other NCDs (Figure 7B). In Ethiopia, an LIC with no reported financial support, >60% of households resorted to extreme coping strategies (Hagos *et al*., 2020; Endale *et al*., 2022).

Some studies reported support from family and friends, government and NGOs: Endale *et al.* (2022) (5%), Tolla *et al*. (2017) (27%), Tsega *et al*. (2021) (13.5%), Okoronko *et al*. (2016) (85%), Kasahun *et al*. (2020) (43%), Opara *et al*. (2021) (48%) and Griffiths *et al*.(2015) (37%).

#### Unmet need for financial reason estimates

None of the studies on the UNFR classified it as an indicator of the financial burden of OOP. Four methods were used to measure UNFR: forgone care (any perceived care not utilized), delayed care, nonadherence to treatment and affordability (Rosenberg *et al*., 2023) (see Supplementary Figure S3).

Different approaches were used to measure affordability. Some studies measured it in terms of days’ wages that a country’s lowest-paid worker needs to spend on a standard course of treatment (Mattila *et al*., 2021; Mensah *et al*., 2021; Ocran Mattila *et al*., 2023). Others asked if treatments were affordable without compromising welfare (Okediji *et al*., 2017; Chianumba *et al*., 2022). One study measured the price of drugs as greater than 5% of monthly earnings (Adeke *et al*., 2022), whereas others measured it using a lack of insurance (Subramanian *et al*., 2018). Unlike forgone, delay or nonadherent to care, there was no uniformity in affordability measurement. Therefore, we removed affordability from our analysis (Supplementary Figures S4 and S5).

The UNFR rate was generally low in UMICs (Supplementary Figure S4). For LwMICs and LICs, the nonadherence rate was very high, particularly for chronic renal diseases (~100%), chronic respiratory diseases (91.9%) and CVD (87.3%) in Nigeria (Akpan *et al*., 2020), Ethiopia (Abegaz *et al*., 2020) and Tanzania (Pallangyo *et al*., 2020), respectively. Delayed and forgone care rates were generally not >85%. High delayed and forgone care rates were observed for cancer and CVD (Supplementary Figure S4). For cancers, delayed and forgone care rates were greater in LICs [for example, in Rwanda (80%) (Kanyamuhunga *et al*., 2015) and Ethiopia (66.3%) (Teshome *et al*., 2021)] than in LwMICs. For CVD, there were only studies from LwMIC, and the delayed and forgone care rate was highest in Cameroon (85.0%) (Dzudie *et al*., 2021) and Ghana (76%) (Nketia-Kyere *et al*., 2017). Many studies reported delayed and forgone care rates for sense organ diseases, but these rates were generally lower than 40% (Abdull *et al*., 2015; Mboni *et al*., 2016; Taryam *et al*., 2020; Tshivhase *et al*., 2020; Bizuneh *et al*., 2021). On average, a higher UNFR was commoner in LIC than in LwMICs (Supplementary Figure S5). Surprisingly, there are no studies on UNFR in the context of the COVID-19 pandemic.

## DISCUSSION

Existing reviews have provided valuable insights into the financial burden of NCDs in SSA. However, these studies focused only on the distribution of NCD-related health costs without comprehensively covering all relevant indicators of financial burden and all important NCDs. Additionally, the increase in the literature since the adoption of the financial risk protection (FRP) indicator as a measure of UHC and the strategic positioning of NCDs in SDG target three necessitates an up-to-date synthesis of the evidence on OOP spending on NCDs. Therefore, this study synthesizes a diverse landscape of 71 studies, providing updated knowledge on the magnitude and evolution of this financial burden in SSA while also highlighting existing evidence gaps.

Most of the studies were without national representativeness. This finding has been reported in another review (Essue *et al*., 2017). This calls for a more comprehensive examination of the financial burden of NCDs. Studies have shown that CHE estimates based on facility-based health expenditure data are usually exaggerated (Essue *et al*., 2017). Our review agrees with that of Mudie *et al*. (2019) in emphasizing the need for additional longitudinal studies to understand the evolution of the financial burden of NCDs, including whether households escape or remain trapped in CHE or impoverishment (Kankeu *et al*., 2013; Kazibwe *et al*., 2021). The small number of studies in our review that used a control group to compare NCD financial burdens would help improve the accuracy of the estimates. This is an important advancement in progress from previous reviews (Kankeu *et al*., 2013; Saksena *et al*., 2014; Essue *et al*., 2017; Jan *et al*., 2018).

The concentration of studies in the West African subregion, particularly Nigeria, highlights the research gaps in other SSA countries, where approximately half have no relevant studies. Njagi *et al*. (2020) also observed a similar predominance of studies from West Africa and Nigeria in the FRP literature (Njagi *et al*., 2018). This dominance might be due to Nigeria having the highest population, being a leader in health research engagement and capacity and being the largest recipient of development assistance for health (DAH) in SSA (Ezeanolue *et al*., 2018; Duncan Knox, 2020; Odunyemi, 2021). This skewed distribution underscores the need for more inclusive, multi-country studies to capture the region’s diverse socioeconomic contexts of FRP.

Moreover, there is bias in the literature towards LwMICs and LICs, as was also found by Essue *et al*. (2017). While it is important to prioritize monitoring in countries with high OOP expenses (Hooley *et al*., 2022; Bolongaita *et al*., 2023), studies in UMICs with stronger FRP could provide insights into effective policies and transferable lessons.

The fluctuating publication rates for NCD research in Africa between 2017 and 2021 likely reflect changing research priorities and funding. Studies suggest that NCD research, particularly FRP research, in Africa, is generally under-prioritized (Tripathy, 2018; Juma *et al*., 2019; Tindana *et al*., 2020), and this, combined with the region’s reliance on donor funding for health research, may have contributed to the observed fluctuations (Grépin *et al*., 2017; Jailobaeva *et al*., 2021). The average shares of all DAH allocated to NCDs in 2018 and 2019 were 0.6% and 1.6%, respectively (Nomura *et al*., 2021). The decrease in DAH between 2017 and 2018, followed by a significant increase between 2020 and 2021 (Duncan Knox, 2020; Micah *et al*., 2023), may have influenced the decrease and spike in the number of studies published during these periods, respectively.

The predominance of three major NCDs—cancer, CVD and diabetes—underscores their importance as the leading cause of mortality in SSA (WHO, 2018a; Gouda *et al*., 2019). However, studies on other NCDs outside the major group are relatively neglected despite the substantial financial burden imposed by some of them (Essue *et al*., 2017; The Lancet Global, 2020; Odunyemi *et al*., 2023). This highlights a crucial gap and emphasizes the need for a balanced representation of both major and lesser-studied NCDs to comprehensively address the economic challenges of NCDs in SSA.

The predominance of studies on UNFR in this review contradicts Rahman *et al*. (2022b), who found no studies examining UNFR for chronic diseases in LMICs. This most probably arose from the more expansive scope of our study. The absence of research on the crowding-out effect of OOP expenditure represents a significant gap, concealing the impact of NCD OOP expenditure on household consumption (Kumara and Samaratunge, 2017; Molla and Chi, 2018). For households with NCDs, studies have shown that even vitally essential expenditures, such as food and education, are not commonly spared (Engelgau *et al*., 2011; Singh *et al*., 2023). Datta *et al.* (2020) showed that OOP spending on blood pressure and diabetes medications crowded out food consumption in all households in Pakistan (Datta *et al*., 2020).

The solitary use of several FRP indicators and the prevalence of UNFR and CHE as sole indicators underscore the need for a more comprehensive approach to capture the financial burden of NCDs in SSA (Moreno-Serra *et al*., 2011).

The dominance of iniquity-prone traditional methods of CHE estimation, particularly budget share and normative food spending methods in our study, is a common finding in the FRP literature (Jan *et al*., 2018; Yerramilli *et al*., 2018; Rahman *et al*., 2022b). This raises concern about the use of these estimates for policy formulation. Moreover, this study, like other studies (Yerramilli *et al*., 2018), reveals an incomplete picture of the severity of CHE because of the sole focus on the headcount ratio (Saksena *et al*., 2014), which is not sensitive to policy monitoring (Koo and Jung, 2022). As noted in other reviews, this lack of rigour in analysis (Yerramilli *et al*., 2018; Rahman *et al*., 2022b) extends to sensitivity analysis and the composite use of indicators, thresholds and equity analysis.

Similarly, the failure to disaggregate impoverishment estimates has significant policy implications. It conceals at-risk and further-impoverished households. Neglecting the latter and the ‘non-spender’ group could lead to an underestimation of IHE (Thomson *et al.*, 2019; Rahman *et al.*, 2022a).

Our study also showed the predominant use of international poverty lines for IHE estimation and inconsistency in the estimates derived from them. This agrees with Ataguba (2021), who showed that the use of absolute international poverty lines instead of relative or national poverty lines has serious policy implications for IHE estimates and could lead to inconsistencies in ranking IHE within and between countries’ socioeconomic groups (Ataguba, 2021).

The infrequent inclusion of children’s withdrawal from school in coping strategy studies in SSA, as also shown in another study (Murphy *et al*., 2019), is an important methodological gap. This issue and the lack of studies on coping strategies in UMICs may indicate a lack of research interest and rigour, which could incorrectly diminish CHE and IHE estimates in the case of UMICs (Flores *et al*., 2008; Wagstaff, 2019).

The multidimensional nature of UNFR is highlighted in our study by the use of multiple measurement methods. However, there was a lack of consistency in the affordability measurements. Additionally, the comparability of UNFR in our study may have been affected by the different recall periods used in the studies, suggesting the need for a standardized approach to UNFR measurements. Unfortunately, there were no studies on NCD-related UNFR during the COVID-19 pandemic. Understanding the level of the financial burden imposed on households with NCDs during the COVID-19 era can inform policy responses aimed at improving access to NCD care and mitigating its economic impact on households.

We observed heterogeneity in CHE estimates across economic groups, NCD types, survey instruments and methodologies. Our study reported high CHE estimates for cancers, CVD and diabetes in SSA. CHE estimates for cancers are particularly high in LwMICs, approaching 100% in some cases, causing a significant financial burden. Cancers have been shown to induce very high CHE, particularly in countries with a low Human Development Index (Doshmangir *et al*., 2021). These findings contradict the findings of Essue *et al*. (2017) and Jan *et al*. (2018), who reported greater CHE for CVD than for cancers in LMICs (Essue *et al*., 2017; Jan *et al*., 2018). While the latter failed to compare estimates based on similar economic groups, estimation methods and thresholds, the former was limited by employing predictive values. On the other hand, the limited number of studies on CHE included in our study might account for these disparities. These findings highlight the urgent need for targeted policies and interventions to address the devastating consequences of CHE from NCDs, particularly cancers in LwMICs.

The finding of higher CHE in LwMICs than in UMICs, indicating a link between a country’s income and economic development and susceptibility to poverty, has been previously reported (Essue *et al*., 2017; Anjorin *et al*., 2022; Rahman *et al*., 2022b; WHO, 2023b). The disparity in CHE could also be due to a higher burden of NCD and healthcare needs in LwMICs and better FRP and health services coverage in UMICs (Ngepah and Ndzignat Mouteyica, 2024).

The unexpectedly lower CHE in LICs than in LwMICs is also well documented (Essue *et al*., 2017; Murphy *et al*., 2020; Rahman *et al*., 2022b; WHO, 2023b). These unexpected findings do not necessarily indicate a better FRP in LICs. A high level of unmet needs, particularly UNFR commonly found in LICs, prevents people from seeking care and invariably affects estimates of CHE and IHE, which only count people who used healthcare. Therefore, in settings of high UNFR, a common occurrence with NCDs, CHE and IHE estimates would appear fictitiously low (Moreno-Serra *et al*., 2011; Thomson *et al*., 2019; Rosenberg *et al*., 2023). This finding highlights the imperative of considering UNFR when interpreting CHE and IHE results.

Our study reveals higher NCD-related CHE in the poorest households across SSA, particularly in LICs and LwMICs. This emphasizes the need for targeted policy interventions to subsidize healthcare costs for low-income families and enhance access to affordable healthcare services (Odipo *et al*., 2024). The smaller disparity between poor and rich households in LICs compared to LwMIC can also be explained by the extensive use of detrimental distressed financing mechanisms and high UNFR in LICs, mostly among the poor. These disparities can have long-term economic impacts, leading to impoverishment and further inequities that affect their overall well-being and economic stability. Insufficient domestic resources for health, overreliance on external aid and lack of access to care, resulting from an inadequate health workforce and poor health infrastructure, particularly in LICs, are largely responsible for the inequality (Ngepah and Ndzignat Mouteyica, 2024). The relatively lower CHE in UMICs is likely due to a 52.5% insurance coverage compared to LwMIC, with just 27.3% coverage (Hooley *et al*., 2022). With more fiscal space, UMICs can subsidize or exempt premiums for the poor, reducing the inequality between the rich and the poor. This variation in CHE between different country income groups highlights the need to consider contextual factors when designing interventions to address high NCD-related CHE.

Our study’s findings of a small disparity between rich and poor households, using budget share, actual food and partial food normative spending methods, align with recent findings (Thomson *et al.*, 2016; Cylus *et al*., 2018; Sas Trakinsky *et al*., 2020). In conformity with our study, the budget share method, the SDG’s official indicator of FRP, has been shown to perform worse in identifying vulnerable populations (Grépin *et al*., 2020; Sas Trakinsky *et al*., 2020). The widespread use of these equity-prone methods in SSA indicates a possible gross underestimation of CHE among the poor.

The fact that health facility-level data showed a smaller disparity between the poor and the rich than household-level data implies that most NCD studies in SSA may have underestimated the existing disparity in CHE.

Like reviews from LMICs (Essue *et al*., 2017; Jan *et al*., 2018; Rahman *et al*., 2022b), our study demonstrates that NCDs drive a high degree of impoverishment in SSA. The degree of limited access to financial resources, such as social safety nets and affordable credit, in LwMICs and LICs, is evident from the use of detrimental coping strategies that could increase people’s vulnerability to future health shocks and impoverishment (Rijal *et al*., 2018; Mirelman *et al*., 2019; Murphy *et al*., 2020).

### Limitations

We acknowledge the limitations of this study, including the limited comparability of our findings across studies due to inadequate data and varying methodological approaches. Although our focus on English language studies may have excluded relevant articles, almost all biomedical studies in SSA, including those in Francophone countries, are published in English (Asubiaro, 2023). The exclusion of multimorbidity in our review limits insights into the comprehensive spectrum of NCD financial burdens. Its inclusion will present methodological and interpretative challenges due to the complexity of the different conditions and their interactions (Kaluvu *et al*., 2022). Our study is also limited by not examining factors associated with the measures of FRP, including the protective effect of health insurance on the financial burden of NCDs, because this is beyond the scope of this study; however, this is best addressed in a systematic review (Eze *et al*., 2022).

## CONCLUSION

The study emphasizes the financial burden of OOP expenditure on NCDs, the methodological gaps in its estimates and associated inequalities. To achieve UHC and SDGs in SSA, key recommendations should address the increasing prevalence of NCDs and implement pro-poor financing schemes and policies. Future research should also address methodological gaps, such as the crowding-out effect, longitudinal trends, the impact of COVID-19, equitable measurement methods and representation across countries and NCD categories. Additionally, future reviews on UNFR are necessary to harmonize the methodologies. Therefore, research capacity, priorities, funding allocation and regional collaboration are crucial.

## Supplementary Material

daae114_suppl_Supplementary_Material
