# Supplementary material for: The financial burden of noncommunicable diseases from out-of-pocket expenditure in sub-Saharan Africa: a scoping review
Source: Health Promot Int. 2024 Sep 17;39(5):daae114. doi: 10.1093/heapro/daae114 (PMC11405128; doi:10.1093/heapro/daae114)
Supplement: daae114_suppl_Supplementary_Material [file daae114_suppl_supplementary_material.doc]

Appendix I

We designed a search strategy using 2Dsearch [1], an online platform that eliminates syntax errors. We applied MeSh subject terms, text words, and their synonyms and pilot tested our search query against the PubMed database. The search strategy was then peer-reviewed by the Murdoch University Library Research Team following the Peer Review of Electronic Search Strategies (PRESS) guidelines [2]. We implemented a definitive search in PubMed and other electronic databases and platforms, including Scopus, ProQuest (Research Library, Dissertations & Theses Global, Econlit, and Health & Medical Collection), and Google Scholar, on 18 August 2023. Using Polyglot Search Translator, we translated the PubMed search strings for use in other databases [3]. The search terms included both generic and specific names of individual NCD categories, the five indicators of financial burden of OOP expenditure discussed earlier, and the names of countries in the SSA, as shown in Supplementary Table 1. Filters were applied to all databases to restrict studies to those published in English between 2015 and 2023 (inclusive). Restrictions were also applied to publication types based on the exclusion criteria. The Google Scholar results were restricted to the first 200 without a language filter.

***Supplementary Table 1: Search Strategy and String Using the PCC (Participants, Concepts and Context) Framework***

| **^¶^No** | **Participant^α^** | **Concept^β^** | **Context^θ^** |
| --- | --- | --- | --- |
|  | Noncommunicable diseases:  *"Noncommunicable diseases"[mh] OR chronic disease*[tw] OR "chronic disease"[mh] OR NCDs[tw] OR non-communicable[tw] OR "non communicable"[tw] OR non-infectious[tw] OR "non infectious"[tw] OR noninfectious[tw]* | Expenditures:  *("out of pocket"[tw] OR "out-of-pocket"[tw] OR "out of pocket health*"[tw] OR "out-of-pocket health*"[tw] OR direct[tw] OR private[tw] OR "private health"[tw] OR impoverish*[tw] OR catastroph*[tw] OR user[tw] OR patient[tw] OR "poverty-induced"[tw]) AND ("health expenditures"[mh] OR "Cost of Illness"[mh] OR spending[tw] OR cost*[tw] OR payment*[tw] OR expens*[tw] OR expenditure*[tw] OR charge*[tw] OR fees[tw]) OR "cost sharing"[tw] OR deductible*[tw] OR coinsurance[tw] OR cost-sharing[tw] OR copayment*[tw] OR co-payment*[tw] OR "Medical Indigency"[mh]* | Sub-Saharan Africa countries:  *"Africa South of the Sahara"[mh] OR "Sub-Sahara* Africa"[tw]* |
|  | Cancers:  *Neoplasms[mh] OR cancer*[tw] OR tumor[tw] OR tumour[tw] OR carcinom*[tw] OR dyspla*[tw] OR hyperpla*[tw]* | Distressed financing:  *"Adaptation, Psychological"[majr] Or "coping mechanism*"[tw] OR "coping behavior"[tw] OR "coping behaviour"[tw] OR coping[tw] OR cope[tw] OR "distress financ*"[tw] OR "coping adaptation*"[tw] OR "hardship financ*"[tw] OR "financial distress"[tw] OR "financial stress"[tw] OR "Financial Stress"[mh] OR "coping strateg*"[tw] OR "coping method*"[tw]* | Central Africa:  *Burundi[tw] OR Cameroon[tw] OR “Central African Republic”[tw] OR Chad[tw] OR Congo[tw] OR “Democratic Republic*  *of the Congo”[tw] OR DRC[tw] OR Congo-Kinshasa[tw] OR “Equatorial Guinea”[tw] OR Gabon[tw] OR “Sao Tome and Principe”[tw]* |
|  | Cardiovascular diseases:  *"Cardiovascular Diseases"[mh] OR heart[tw] OR cardiovascular[tw] OR angina[tw] OR fibrillat*[tw] OR cardiomyopath*[tw] OR cardiac failure*[tw] OR heart failure*[tw] OR cardia*[tw] OR hypertensi*[tw] OR myocard*[tw] OR stroke[tw] OR vascular[tw] OR ventricular[tw] OR aneurysm[tw] OR ischaem*[tw] OR atrial[tw] OR infarct*[tw] OR ischem*[tw] OR cerebrovascular[tw] OR coronary[tw] OR "rheumatic heart"[tw] OR cerebral vasc*[tw]* | Crowd out effect:  *"consumption insurance"[tw] OR "income smoothing"[tw] OR "consumption displacement*"[tw] OR "crowd* out"[tw] OR crowd-out[tw] OR "consumption smoothing"[tw]* | Eastern Africa: *Comoros[tw] OR Eritrea[tw] OR Ethiopia[tw] OR Kenya[tw] OR Madagascar[tw] OR Mauritius[tw] OR Rwanda[tw] OR*  *Seychelles[tw] OR Somalia[tw] OR “South Sudan”[tw] OR Sudan[tw] OR Uganda[tw] OR “Tanzania[tw]* |
|  | Diabetes:  *"Diabetes Mellitus"[mh] OR diabetes[tw] OR hyperglycaemia[tw] OR hyperglycemia[tw] OR "glucose intolerance"[tw]* | Unmet need for financial reason:  *"unaffordable medical care"[tw] OR "unmet healthcare need*"[tw] OR "unmet need*"[tw] OR "unmet health need*"[tw] OR "cost-related unmet need*"[tw] OR "forgo* healthcare"[tw] OR "unmet health care need*"[tw] OR "unaffordable health care"[tw] OR "unaffordable care"[tw] OR "financial barrier*"[tw] OR "financial reason*"[tw] OR "forgo* health care"[tw] OR "forgo* care"[tw] OR "economic reason*"[tw]* | Southern Africa: *Angola[tw] OR Botswana[tw] OR Eswatini[tw] OR Lesotho, Malawi[tw] OR Mozambique[tw] OR Namibia[tw] OR “South*  *Africa” OR Zambia[tw] OR Zimbabwe[tw]* |
|  | Chronic respiratory diseases:  *"chronic obstructive airway"[tw] OR COAD*[tw] OR COPD*[tw] OR "chronic obstructive respiratory"[tw] OR "chronic obstructive pulmonary"[tw] OR "obstructive respiratory"[tw] OR "obstructive airway"[tw] OR "obstructive pulmonary"[tw] OR "chronic respiratory"[tw] OR "chronic airway"[tw] OR "chronic pulmonary"[tw] OR asthma[tw] OR emphysema[tw] OR "chronic bronchitis"[tw] OR bronchiectasis[tw]* | Financial burden:  *(financ*[tw] OR economic*[tw]) AND (burden*[tw] OR challeng*[tw] OR pressure*[tw] OR hardship*[tw] OR cost*[tw] OR impact*[tw] OR effect*[tw] OR consequence*[tw] OR outcome*[tw] OR toxicit*[tw] OR risk*[tw])* | Western Africa: *Benin[tw] OR “Burkina Faso”[tw] OR ”Cabo Verde”[tw] OR “Côte d’Ivoire”[tw] OR "Ivory Coast"[tw] OR Gambia[tw] OR Ghana[tw] OR Guinea[tw] OR “Guinea Bissau”[tw] OR Liberia[tw] OR Mali[tw] OR Mauritania[tw] OR Niger[tw] OR Nigeria[tw] OR Senegal[tw] OR “Sierra Leone”[tw] OR Togo[tw]* |
|  | Mental Disorders:  *"Mental Disorders"[mh] OR depression[tw] OR schizophren*[tw] OR obsessive-compulsive[tw] OR compulsive[tw] OR obsess*[tw] OR anorex*[tw] OR bulim*[tw] OR insomni*[tw] OR phobi*[tw] OR anxiet*[tw]* |  |  |
|  | Neurological disorders:  *"Nervous System Diseases"[mh] OR epilepsy[tw] OR convuls*[tw] OR seizure*[tw] OR migrain*[tw] OR dement*[tw] OR neurologic*[tw] OR Parkinson*[tw] OR headache[tw] OR "head ache"[tw] OR sclerosis[tw] OR cephalg*[tw] OR encephal*[tw] OR hydrocephalus[tw]* |  |  |
|  | Chronic renal diseases:  *"Kidney Diseases"[mh] OR renal[tw] OR kidney*[tw] OR glomer*[tw] OR nephr*[tw]* |  |  |
|  | Musculoskeletal diseases:  *"Musculoskeletal Diseases"[mh] OR musculoskeletal[tw] OR arthrit*[tw] OR osteoarthrit*[tw] OR gout*[tw] OR rheumat*[tw] OR spondyl*[tw]* |  |  |
|  | Heamatological disorders:  *"Hematologic Diseases"[mh] OR hematolog*[tw] OR blood[tw] OR thromb*[tw] OR anaem*[tw] OR anem*[tw] OR anaemic[tw] OR anemic[tw] OR haemoglobin*[tw] OR hemoglobin*[tw] OR haemophilia*[tw] OR hemophilia*[tw] OR erythro*[tw] OR "sickle cell"[tw] OR pancytopen*[tw] OR polycythem*[tw] OR leukem*[tw] OR lympho*[tw] OR auntoimmun*[tw] OR aunto-immun*[tw]* |  |  |
|  | Sense organ diseases:  *"Sense Organs"[mh] OR glaucoma[tw] OR cataract[tw]* |  |
|  | Gastrointestinal diseases:  *"Gastrointestinal Diseases"[mh] OR "peptic ulcer"[tw] OR gastric ulcer*[tw] OR duoden* ulcer*[tw] OR hernia[tw] OR Crohn's[tw] OR stomach ulcer*[tw] OR "inflammatory bowel"[tw]* |  |
|  | ^§^Exclusions:  *smoking[tw] OR inactivity[tw] OR "air pollution"[tw] OR diet[tw] OR food nutrition*[tw] OR obesity[tw] OR overweight[tw] OR metabolic*[tw] OR comorbidit*[tw] OR co-morbidit*[tw] OR multi-morbidit*[tw] OR multimorbidit*[tw] OR prevent*[tw]* | ^§^Exclusions:  *cost effectiv*[tw] OR cost-effectiv*[tw] OR costeffectiv*[tw] OR clinical effectiv*[tw]* |  |
| *Notes*: ^α^The grouping of the search terms for the categories of noncommunicable diseases (and their synonyms and MeSH Terms) included in this study.  ^β^The grouping of the search terms for the concepts of financial burden of out-of-pocket expenditure (and their synonyms and MeSH Terms) included in this study.  ^θ^The list of the 48 countries (and their synonyms) of the sub-Saharan African region, as defined by the World Bank. Countries were grouped into four regions using the African Union classification.  **^¶^**Except the exclusion items, we combine terms under the same item with Boolean term *OR* and join the group to other category using the Boolean term *AND.*  ^§^Each exclusion item was combined with the rest of its category using the boolean term *NOT.* | | | |


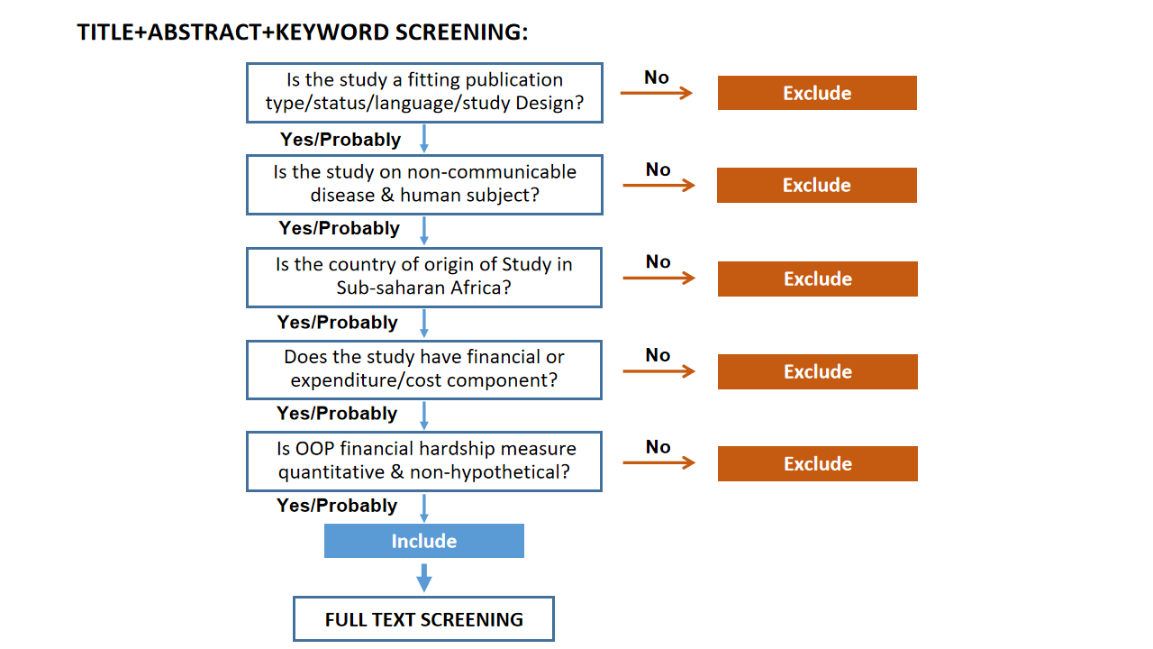


***Supplementary Figure 1:* The Decision Tree for Title and Abstract Screening of Studies**


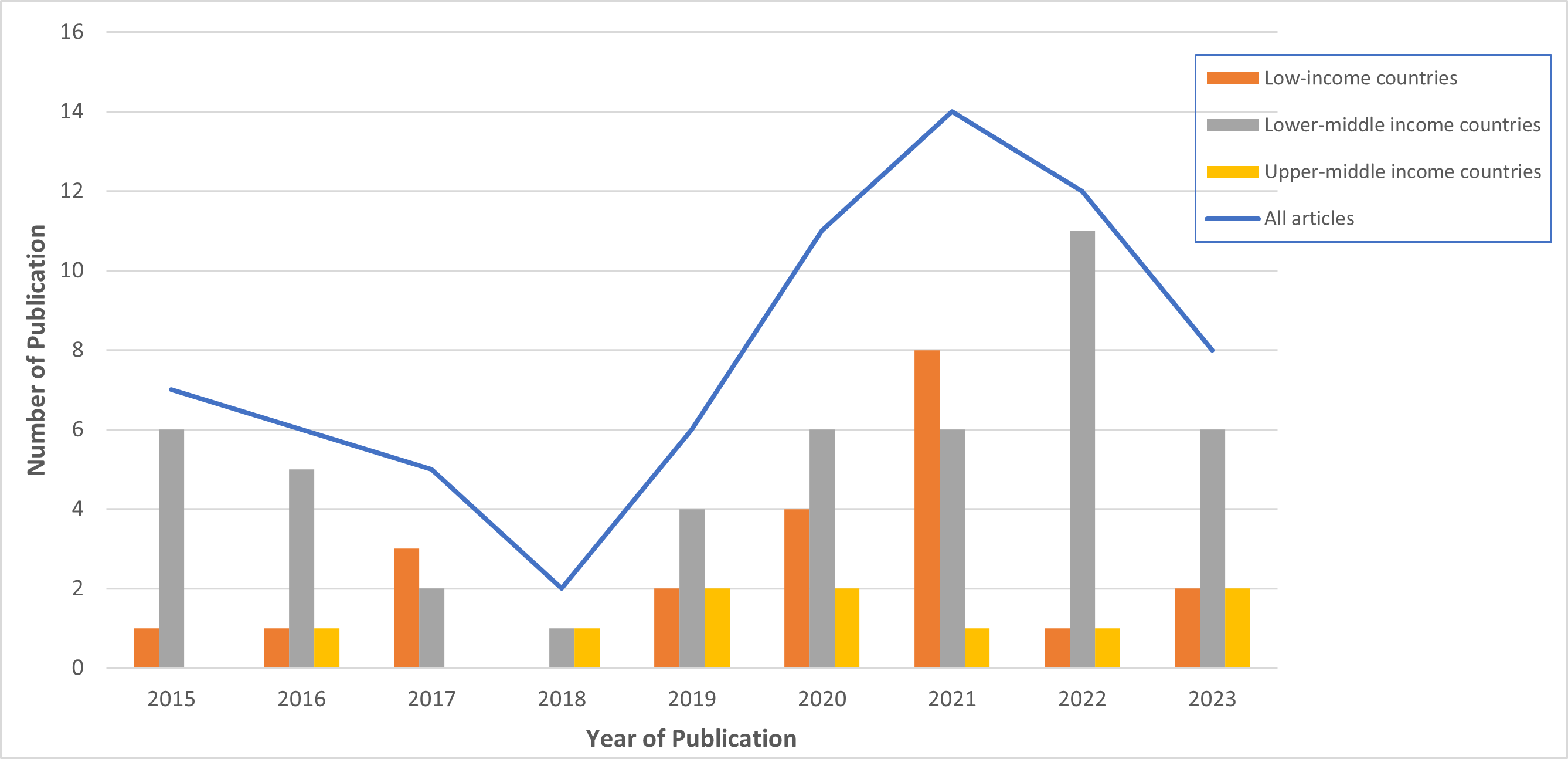


***Supplementay Figure 2:* Annual distribution of studies by country's economic group**

Note: Multicountry studies covering multiple economic groups are counted for each economic group so that the total number of studies depicted in the figure typically exceeds the number of included studies in the review.

***Supplementary Table 2:* Distribution of the studies by method, threshold, intensity and type of equity analysis used for catastrophic health expenditure estimation**

| **CHE Estimation Method** | **Single Country Studies, *n* (%)** | | | **Multicountry Studies, *n* (%)** | **All Studies, *n* (%)** |
| --- | --- | --- | --- | --- | --- |
|  | **LIC** | **LwMIC** | **UMIC** |  |  |
| Budget share only method | 5 (45.4) | 4 (19.0) | 0 (0.0) | 0 (0.0) | 9 (23.1) |
| Actual food expenditure method only | 1 (9.1) | 2 (9.5) | 0 (0.0) | 1 (20.0) | 4 (12.3) |
| Partial Normative food expenditure method only | 0 (0.0) | 5 (23.8) | 1 (50.0) | 1 (20.0) | 7 (17.9) |
| WHO Europe method only | 0 (0.0) | 0 (0.0) | 0 (0.0) | 1 (20.0) | 1 (2.6) |
| Ataguba method only | 0 (0.0) | 1 (4.8) | 0 (0.0) | 0 (0.0) | 1 (2.6) |
| Any of the older capacity-to-pay methods (Actual food and partial food normative) together | 1 (9.1) | 1 (4.8) | 0 (0.0) | 2 (40.0) | 4 (10.3) |
| Budget share with older capacity-to-pay method | 2  (18.2) | 3 (14.3) | 0 (0.0) | 1 (20.0) | 6 (15.4) |
| Budget share with WHO Europe method | 0 (0.0) | 1 (4.8) | 0 (0.0) | 1 (20.0) | 2 (5.1) |
| Budget share with Ataguba | 0 (0.0) | 0 (0.0) | 0 (0.0) | 0 (0.0) | 0 (0.0) |
| Any of the older capacity to pay with WHO Europe method | 0 (0.0) | 1 (4.8) | 0 (0.0) | 0 (0.0) | 1 (2.6) |
| Any older capacity to pay method with Ataguba method | 0 (0.0) | 0 (0.0) | 1 (50.0) | 0 (0.0) | 1 (2.6) |
| Total | 11 (100.0) | 21 (100.0) | 2 (100.0) | 5 (100.0) | 39 (100.0) |
|  |  |  |  |  |  |
| **Intensity of CHE** |  |  |  |  |  |
| Headcount ratio | 7 (70.0) | 16 (88.9) | 1 (100) | 4 (100.0) | 28 (84.8) |
| Mean/Median positive gap | 2 (20.0) | 1 (5.5) | 0 (0.0) | 0 (0.0) | 3 (9.1) |
| (Mean) positive overshoot | 1 (10.0) | 1 (5.5) | 0 (0.0) | 0 (0.0) | 2 (6.1) |
| Negative overshoot | 0 (0.0) | 0 (0.0) | 0 (0.0) | 0 (0.0) | 0 (0.0) |
| Total | 10 (100) | 18 (100) | 1 (100.0) | 4 (100.0) | 33 (100.0) |
|  |  |  |  |  |  |
| **Threshold of CHE** |  |  |  |  |  |
| Single fixed | 5 (62.5) | 8 (50) | 0 (0.0) | 3 (75) | 16 (55.2) |
| Multiple fixed | 3 (37.5) | 7 (43.7) | 0 (0.0) | 1 (25) | 11 (37.9) |
| Rank-dependent (Ataguba) | 0 (0.0) | 1 (6.3) | 1 (100) | 0 (0.0) | 2 (6.9) |
| Total | 8 (100) | 16 (100) | 1 (100) | 4 (100) | 29 (100) |
|  |  |  |  |  |  |
| **Equity Analysis of CHE** |  |  |  |  |  |
| Wealth Quintile only | 5 (100.0) | 11 (91.7) | 1 (50.0) | 2 (100.0) | 19 (90.5) |
| Gini index (coefficient)/Lorenz curve | 0 (0.0) | 0 (0.0) | 0 (0.0) | 0 (0.0) | 0 (0.0) |
| Concentration index/curve | 0 (0.0) | 1 (8.3) | 1 (50.0) | 0 (0.0) | 2 (9.5) |
| Kakwani index | 0 (0.0) | 0 (0.0) | 0 (0.0) | 0 (0.0) | 0 (0.0) |
| Total | 5 (100.0) | 12 (100.0) | 2 (100.0) | 2 (100.0) | 21 (100.0) |
| Notes: *CHE* means catastrophic health expenditure.  *LIC* means low-income country, *LwMIC* means lower-middle-income country, and *UMIC* means upper-middle-income country. | | | | | |

***Supplementary Table 3:* Distribution of the studies by method and depth of analysis of impoverishing health expenditure estimation**

| **IHE Estimation Method** | **Single Country Studies, *n* (%)** | | | **Multi-country Studies, *n* (%)** | **All Studies, *n* (%)** |
| --- | --- | --- | --- | --- | --- |
|  | **LIC** | **LwMIC** | **UMIC** |  |  |
| Traditional Method | 2 (100.0) | 5 (83.3) | 1 (100.0) | 0 (0.0) | 8 (80.0) |
| WHO Europe Method | 0 (0.0) | 1 (16.7) | 0 (0.0) | 1 (100.0) | 2 (20.0) |
| Total | 2 (100.0) | 6 (100.0) | 1 (100.0) | 1 (100.0) | 10 (100.0) |
|  |  |  |  |  |  |
| **Intensity of Impoverishment** |  |  |  |  |  |
| Headcount ratio (Proportion of households impoverished) | 2 (50.0) | 6 (85.7) | 1 (100) | 1 (50.0) | 10 (71.4) |
| Impoverishment gap | 1 (25.0) | 0 (0.0) | 0 (0.0) | 0 (0.0) | 1 (7.1) |
| Normalized poverty gap/Poverty gap index | 1 (25.0) | 0 (0.0) | 0 (0.0) | 0 (0.0) | 1 (7.1) |
| Intensity of impoverishment | 0 (0.0) | 0 (0.0) | 0 (0.0) | 0 (0.0) | 0 (0.0) |
| Immiserised (Further impoverished) households | 0 (0.0) | 1 (14.3) | 0 (0.0) | 1 (50.0) | 2 (14.3) |
| Total | 4 (100) | 7 (100) | 1 (100.0) | 2 (100.0) | 14 (100.0) |
|  |  |  |  |  |  |
| **Equity Analysis of Impoverishment** |  |  |  |  |  |
| Wealth Quintile only | 0 (0.0) | 3 (100.0) | 0 (0.0) | 0 (0.0) | 3 (100.5) |


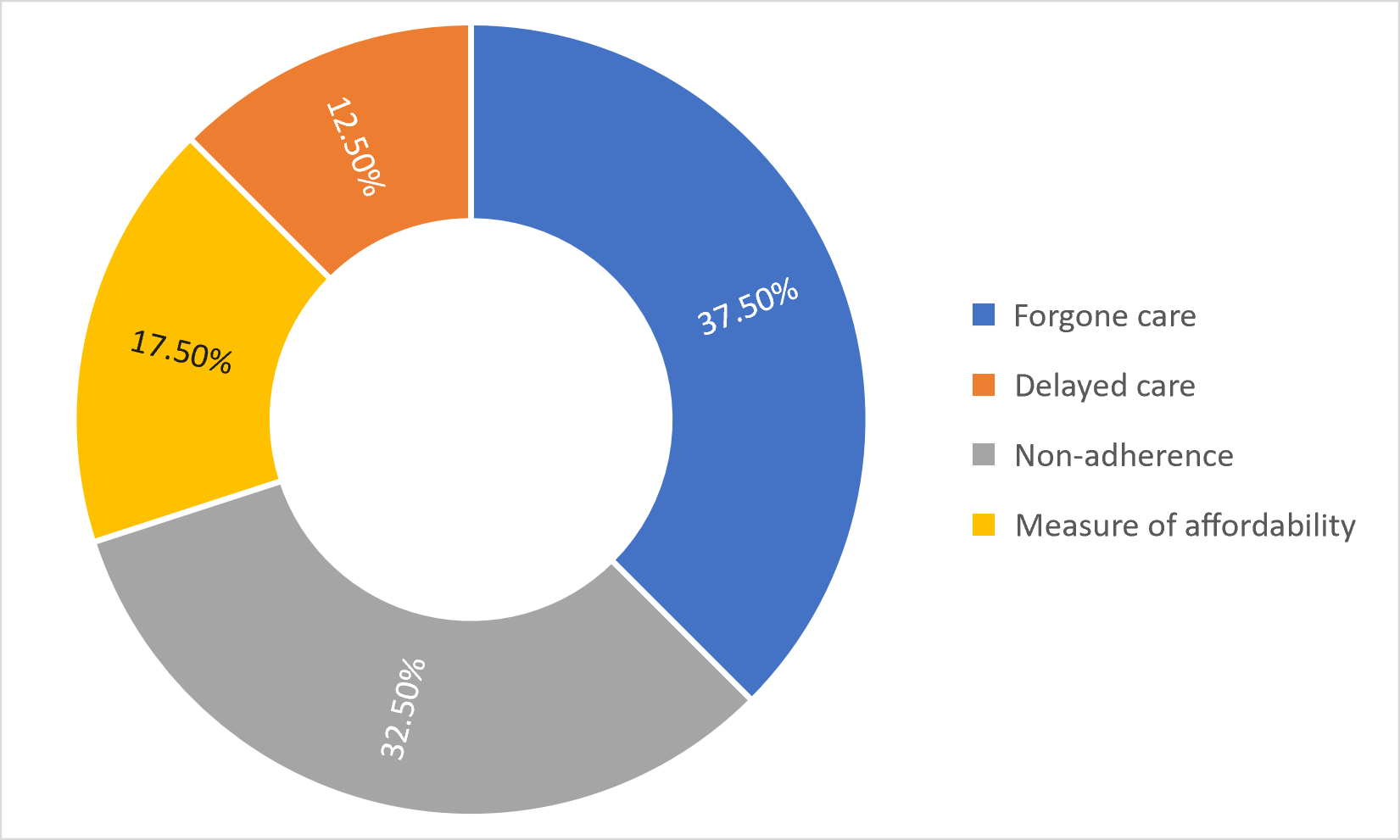


***Supplementary Figure 3:* Distribution of Methods of Assessing Unmet Need for Financial Reason**


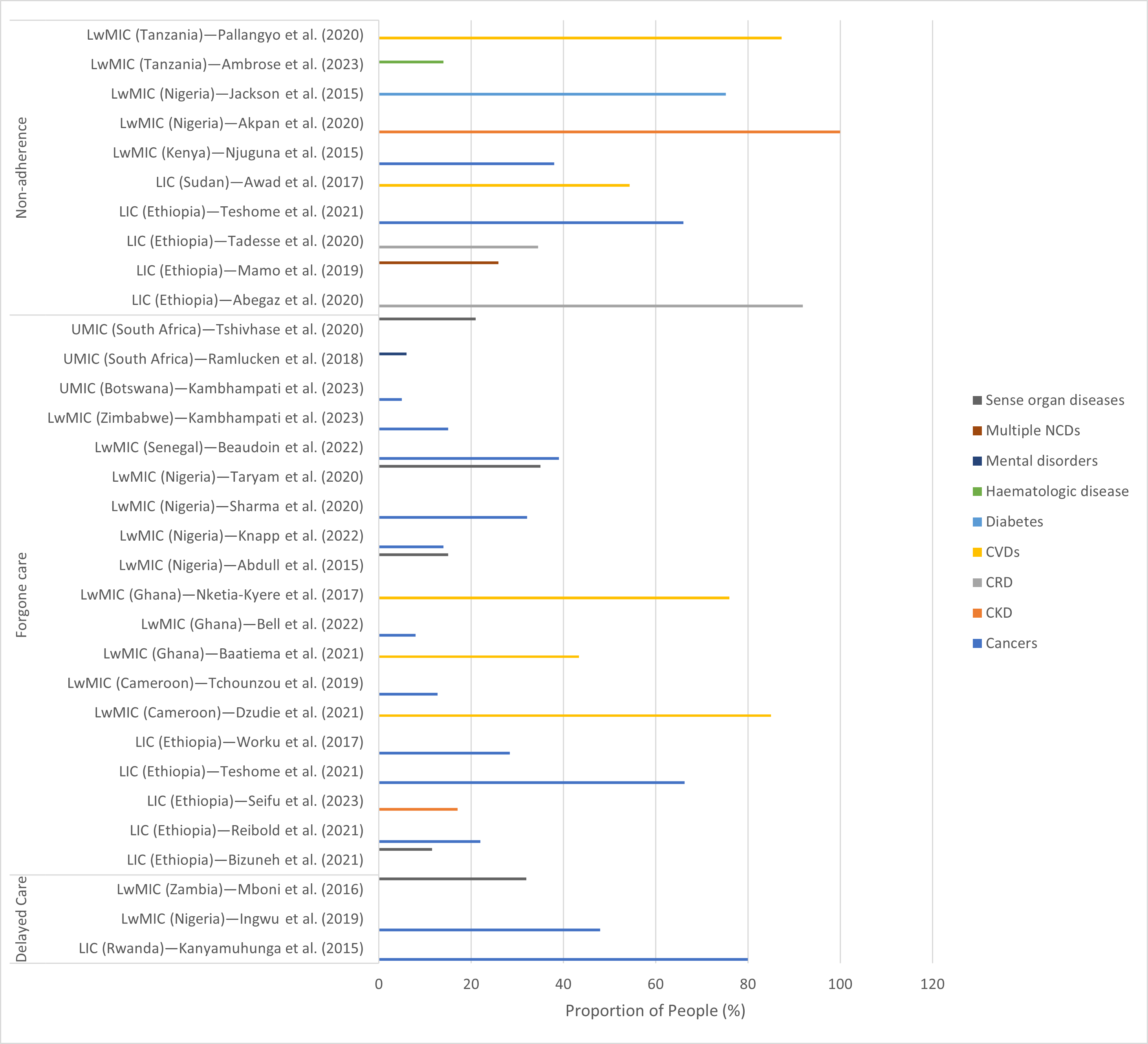


***Supplementary Figure 4:* Proportion of people with unmet need for financial reasons (study-specific) by estimation type and noncommunicable disease category in Sub-Saharan Africa countries**

Note: Studies that used “Affordability” to measure unmet need were excluded because they rarely estimate unmet need as a proportion.

Multi-country studies without country-specific data were also excluded.

*LIC* low-income country, *LwMIC* lower-middle-income country, *UMIC* upper-middle-income country, *NCDs* non-communicable diseases, CVDs cardiovascular disease, *CRD* chronic respiratory diseases, and *CKD* chronic renal diseases


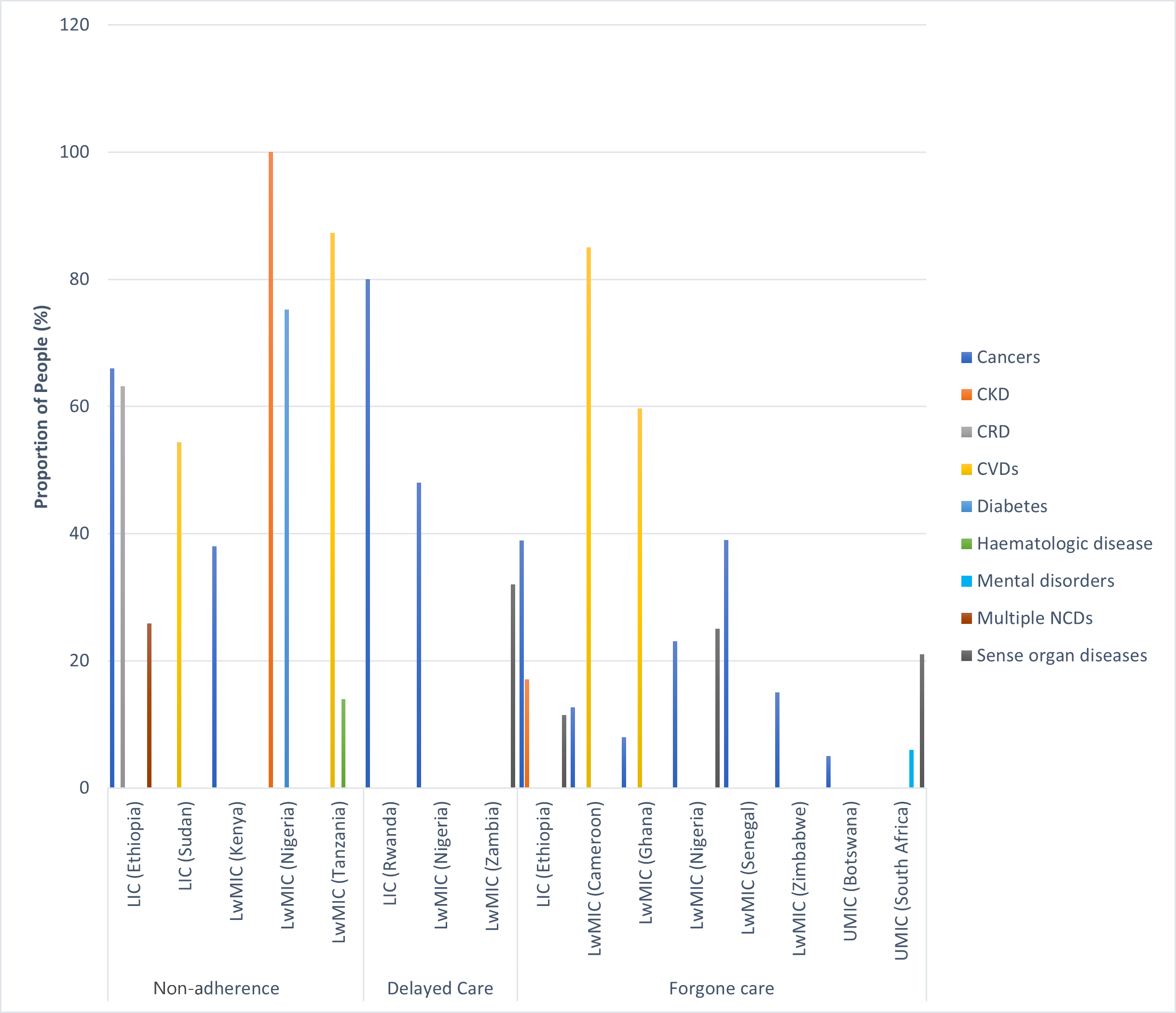


***Supplementary Figure 5:* Mean proportion of people with unmet need for financial reasons by estimation type and noncommunicable disease category in Sub-Saharan Africa countries**

Note: Studies that used “Affordability” to measure unmet needs were excluded because they rarely estimate unmet need as a proportion.

*LIC* low-income country, *LwMIC* lower-middle-income country, *UMIC* upper-middle-income country, *NCDs* non-communicable diseases, CVDs cardiovascular disease, *CRD* chronic respiratory diseases, and *CKD* chronic renal diseases

##### Reference:
